# Green synthesis of new adamantane-containing dihydropyrimidine derivatives as potential anticancer agents†

**DOI:** 10.1039/d5ra00284b

**Published:** 2025-03-13

**Authors:** Adeleh Moshtaghi Zonouz, Mina Abkar Aras, Nahideh Jafari, Zahra Rezaei, Hamed Hamishehkar

**Affiliations:** a Department of Chemistry, Faculty of Science, Azarbaijan Shahid Madani University Tabriz Iran adelehmz@yahoo.com ac.moshtaghi@azaruniv.ac.ir; b Faculty of Chemical and Petroleum Engineering, University of Tabriz Tabriz Iran; c Drug Applied Research Center, Tabriz University of Medical Sciences Tabriz Iran hamishehkarh@tbzmed.ac.ir Hamishehkar.hamed@gmail.com

## Abstract

Despite significant progress in cancer treatment, cancer remains a major focus of research due to medication resistance and side effects. In this study, bioactive adamantane-containing dihydropyrimidine (DHPM) derivatives were synthesized through the multi-component Biginelli reaction of *N*-(adamant-1-yl)acetoacetamide, benzaldehyde derivatives, and thiourea in the presence of trifluoroacetic acid (TFA, 2 mol%) as catalyst under solvent free conditions. This method provides an effective and significantly improved modification of the original Biginelli reaction in terms of yield and reaction time. The synthesized DHPM derivatives were subjected to cytotoxicity screening against the A-549 human non-small cell lung cancer (NSCLC) cell line to evaluate their effects on cell growth inhibition. MTT cytotoxicity assay was used to determine IC_50_ values. Among the target analogs, IIb, IIj, IId, and IIg demonstrated the best activity with IC_50_ values of 1.03, 8.36, 10.38, and 16.04 μg mL^−1^, respectively. Additionally, we assessed the possible mechanisms for cell growth inhibition and induction of apoptotic cell death using the DAPI and Annexin V-FITC staining. The average percentages of apoptotic cells were 21.35%, 28.35%, 32.73, and 43.33% for IIg, IId, IIj, and IIb treatment groups, respectively. These results suggest that the synthesized adamantane-containing dihydropyrimidines can be considered as encouraging molecules for further drug development as anticancer agents.

## Abstract 2

Adamantane-containing dihydropyrimidine derivatives were synthesized under solvent-free conditions and their cytotoxic activities were evaluated against the A-549 lung cancer cells.

## Graphical abstract

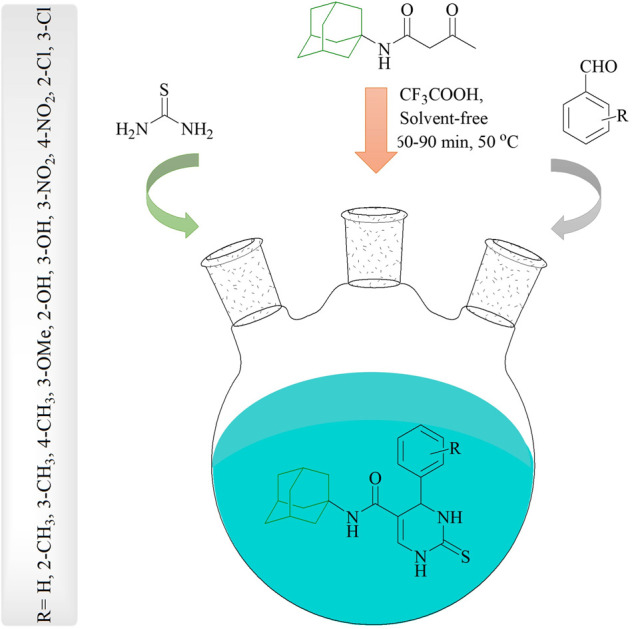

## 1. Introduction

Cancer is a serious condition associated with complex biological processes in which a group of cells proliferates abnormally. The disease has been recognized as an important global health problem.^1^ The number of cancer-related deaths worldwide is expected to increase to 12 million annually by 2030. Despite the enormous progress in cancer treatment over the past few decades, it remains a major area of research due to medication resistance and drug side effects. Hence, it is essential to conduct research on drug development for cancer treatment. Heterocyclic structures remain interesting synthesis candidates due to their significant biological properties. Particularly, dihydropyrimidine (DHPM) derivatives are well recognized for their extensive biological functions.^2^ The DHPM molecules are structural analogs of Monastrol with important biological characteristics such as antiviral,^3^ anticancer,^4^ antibacterial,^5^ and calcium channel function modulation.^6^ Pharmaceutical scientists have used Monastrol to formulate new chemotherapy drugs based on the DHPM structure or its modification with other substitutions. Numerous investigations have shown that DHPM derivatives like Monastrol inhibit human kinesin Eg5, leading to apoptosis.^7^ However, some studies showed other targets, such as centrin, calcium channels, and topoisomerase I for DHPM molecules. In nucleic acids, three types of nucleobases are pyrimidine derivatives, which include cytosine in DNA and RNA, uracil in RNA, and thymine in DNA.^8^ Hence, designing and developing DHPM derivatives is a stimulating focus for synthetic organic chemistry. Solvent-free and catalyst-free green techniques have been reported as a method to produce DHPM derivatives and their synthesis. In a study, Pramanik *et al.* used common fruit juice to develop an efficient, sustainable, and environmentally friendly method for preparing DHPM derivatives at room temperature.^9^ Bhatewara *et al.* produced DHPM using microwave irradiation and assessed their antibacterial characteristics.^10^ Also Saleem Farooq *et al.* developed a simple, efficient, cost-effective, and green approach for the synthesis of dihydropyrimidine derivatives *via* the Biginelli reaction.^11^ In another study, Morvarid Najjar *et al.* synthesized dihydropyrimidinone derivatives by a GQDs-based magnetically nanocatalyst under solvent-free conditions.^12^ A comparable study recognized the adamantane moiety as a crucial pharmacophore in several pharmacologically active drugs. The incorporation of the lipophilic adamantyl moiety into several bioactive molecules enhanced lipophilicity, improving bioavailability and controlling therapeutic efficiency.^13^ In our previous study, some adamantane-containing DHPM derivatives have been synthesized using a three-component Biginelli reaction of *N*-(adamant-1 yl)acetoacetamide, benzaldehyde derivatives and thiourea in acetonitrile in the presence of trifluoroacetic acid (TFA, 10 mol%) as a catalyst.^14^

Accordingly, this study aimed to report a green synthesis for adamantane-containing DHPM derivatives under solvent-free conditions. To this end, we synthesized DHPM derivatives through the Biginelli reaction of *N*-(adamant-1-yl)acetoacetamide, benzaldehyde derivatives, thiourea, and TFA (2 mol%) as a catalyst. This technique provides an effective improved modification of the original Biginelli reaction in terms of yield and short reaction times under solvent-free conditions. The prepared compounds have been characterized using FT-IR, ^1^H and ^13^C NMR spectra, and elementary analyses. Finally, the cytotoxic activity of the DHPM derivatives was assessed against the A-549 lung cancer cells.

## 2. Results and discussion

### 2.1 Chemistry

**Scheme 1:**
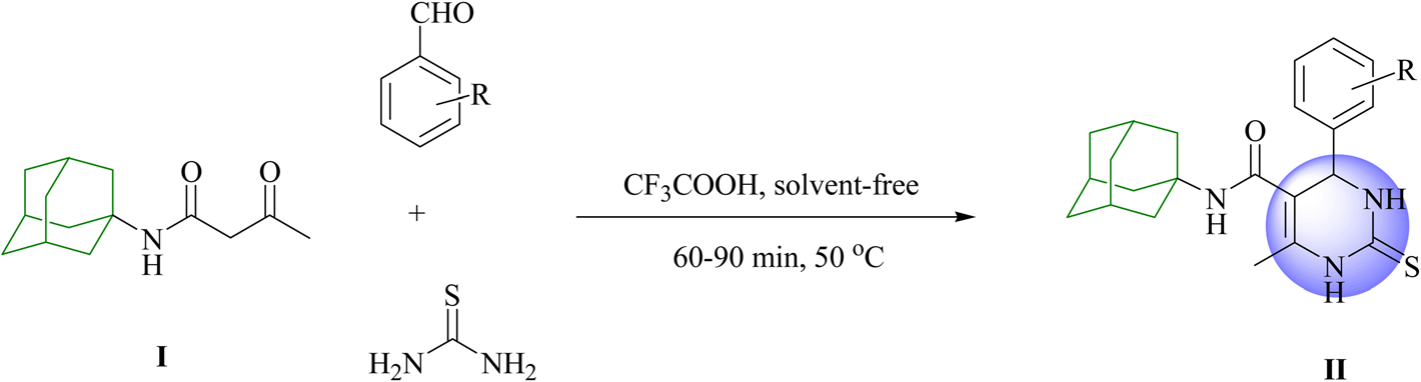
Synthesis of 5-(1-adamantylcarbamoyl)-6-methyl-4-aryl-3,4-dihydropyrimidin-2(1*H*)-thiones II*via* Biginelli reaction.

Recently, we reported the one-pot three-component Biginelli procedure for the preparation of adamantane-containing DHPM derivatives using *N*-(adamantan-1-yl)acetoacetamide, benzaldehyde derivatives, and thiourea in CH_3_CN under reflux conditions for 36–42 h in the presence of TFA (10 mol%).^14^ Nowadays, one of the ultimate goals and challenges in ecofriendly synthetic methodologies is the green process for the preparation of biologically active substances from small and commercially available compounds. Many organic solvents and catalysts are potentially toxic to the ecosystem. Therefore, their use should be minimized or even be replaced with green alternative solvents and catalysts. Accordingly, in this research we prepared the potentially valuable nitrogen-containing heterocyclic molecules and described Biginelli-type synthesis of adamantane-containing DHPMs through one-pot three-component condensation of *N*-(adamant-1-yl)acetoacetamide (I), benzaldehyde derivatives, and thiourea in the presence of a catalytic amount of TFA (2 mol%) under solvent-free conditions (Scheme 1). *N*-(Adamantan-1-yl)acetoacetamide (I) was prepared by nucleophilic amidation of ethyl acetoacetate with 1-adamantylamine in the presence of a catalytic amount of triethylamine (5 mol%) in refluxing toluene under an argon atmosphere. First, the reaction of benzaldehyde (1 equiv.), *N*-(adamantan-1-yl)acetoacetamide (1 equiv.), and thiourea (1 equiv.) was investigated under solvent-free conditions without any catalyst at room temperature. This condition did not afford the desired product. Then, reaction was performed in the presence of catalyst. However, using catalyst such as CF_3_COOH at room temperature did not afford the desired product. Therefore, the reaction was performed at different temperatures. The best result was obtained when the reaction was carried out at 50 °C. This process was improved by modulating reaction temperature, reaction time, and chemical proportions under solvent-free conditions.

Upon further evaluation, the enhanced yields and optimal reaction times were achieved when using TFA (2 mol%), *N*-(adamant-1-yl)acetoacetamide (1 mmol), 2-methyl benzaldehyde (1 mmol), and thiourea (1 mmol) under solvent-free conditions at 50 °C, resulting in the synthesis of DHPM IIb in 85% yield within 60 minutes. A series of different DHPM analogs IIa–k were then synthesized using these optimized conditions (Scheme 1 and Table 1).

**Table 1:** Synthesis of pyrimidines II incorporating an adamantane moiety

Entry	R	Product	Time (min)	Yield (%)	MP (°C)
1	H	IIa	60	83	238
2	2-Me	IIb	60	85	258
3	3-Me	IIc	85	81	256
4	4-Me	IId	85	70	230
5	3-OMe	IIe	70	74	256–259
6	2-OH	IIf^a^	90	75	>250
7	3-OH	IIh	90	80	181–185
8	3-NO_2_	IIi	90	60	>270
9	4-NO_2_	IIg	90	68	216–218
10	2-Cl	IIj	60	67	184–186
11	3-Cl	IIk	90	50	238

a IIf is 8-oxa-10,12-diazatricyclo [7.3.1.0^2,7^] tridecatriene derivative.

The obtained products were characterized by IR, ^1^H NMR and ^13^C NMR. For example, the FT-IR spectrum of 5-(1-adamantylcarbamoyl)-6-methyl-4-nitrophenyl-3,4-dihydropyrimidin-2(1*H*)-thione (IIg) confirmed the presence of the functional groups NH at 3369 cm^−1^ and 3185 cm^−1^, and the amide carbonyl (NH–C=O) as a strong absorption band around 1619 cm^−1^ and C=S at 1250 cm^−1^. The ^1^H NMR spectrum of IIg displayed adamantane protons at 1.55 (s, 6H, Ad), 1.83 (s, 6H, Ad), 1.93 (s, 3H, Ad) ppm, the CH-4 proton at 5.29 ppm, and the three NH protons at 7.23, 9.31, and 9.89 ppm. The ^13^C NMR spectrum further confirmed the product by the presence of an amide carbonyl signal at 165.6 and C=S at 174.8 ppm. The presence of C=C signals at 107.8 and 150.6 ppm was another reason for confirming the formation of this product. Similar to the our previous research,^14^ with *ortho* hydroxy-substituted aromatic aldehyde, salicylaldehyde, the expected product is not a simple DHPM. However, after the domino Biginelli condensation/Michael addition reaction, the 13-(1-adamantylcarbamoyl)-9-methyl-11-thioxo-8-oxa-10,12-diazatricyclo[7.3.1.0^2,7^]trideca-2,4,6-triene derivative (IIf) was obtained. The intramolecular Michael addition might be due to the steric proximity of the *ortho* OH substituent of the aromatic ring to the C-6 carbon of the pyrimidine ring.

### 2.2 Biological evaluation

#### 2.2.1 Evaluation of *in vitro* cytotoxic effects

**Fig. 1:**
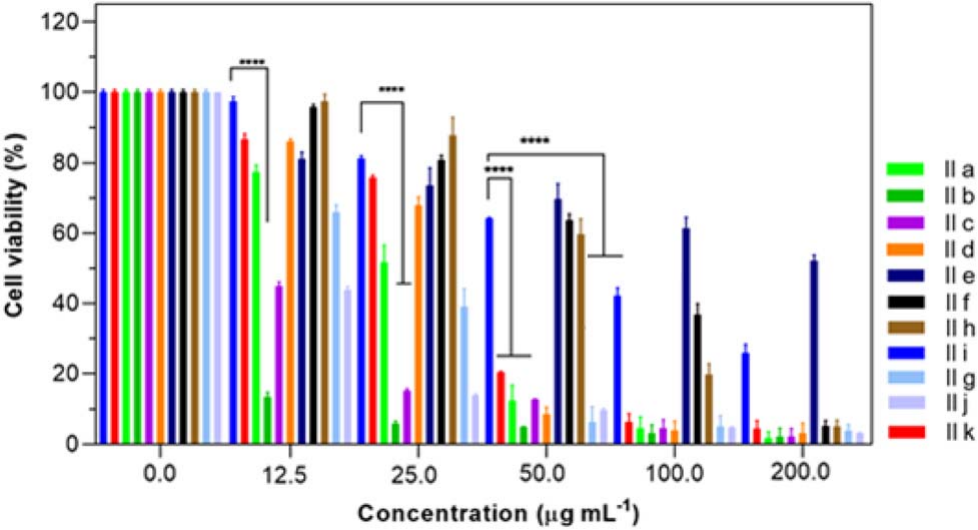
Cell viabilities of non-small-cell lung cancer cell lines A-549 with different treatment groups for 48 h. ****P < 0.0001 (*n* = 3).

Using the MTT assay, the obtained DHPM derivatives IIa–k were evaluated for their cytotoxicity against A-549 cancer cell lines.^15^ Interestingly, the investigated compounds demonstrated excellent to moderate cytotoxicity on the A-549 cancer cell lines in a dose- and time-dependent cytotoxicity manner (Fig. 1). Among these, the compounds 5-(1-adamantylcarbamoyl)-6-methyl-4-(2-methylphenyl)-3,4-dihydropyrimidin-2(1*H*)-thione (IIb), 5-(1-adamantylcarbamoyl)-6-methyl-4-(2-chlorophenyl)-3,4-dihydropyrimidin-2(1*H*)-thione (IIj), 5-(1-adamantylcarbamoyl)-6-methyl-4-(4-methylphenyl)-3,4-dihydropyrimidin-2(1*H*)-thione (IId), and 5-(1-adamantylcarbamoyl)-6-methyl-4-(4-nitrophenyl)-3,4-dihydropyrimidin-2(1*H*)-thione (IIg) had excellent cytotoxic activity with IC_50_ values of 1.03, 8.36, 10.38, and 16.04 μg mL^−1^, respectively (Table 2). As can be seen, the compound 5-(1-adamantylcarbamoyl)-6-methyl-4-(2-methylphenyl)-3,4-dihydropyrimidin-2(1*H*)-thione (IIb) shows even better cytotoxicity manner than the common anticancer drug (doxorubicin).

**Table 2:** IC_50_ μg mL^−1^ of DHMP treatment groups against A-549 cell lines

Compounds	IIa	IIb	IIc	IId	IIe	IIf	IIg	IIh	IIi	IIj	IIk
IC_50_ (μg mL^−1^)	19.82	1.03	16.94	10.38	41.80	91.56	16.04	64.89	79.59	8.36	24.23

Substitution of a methyl group on the phenyl ring displayed significant cytotoxicity against A-549 cancer cell lines with IC_50_ values of 1.03 and 10.38 μg mL^−1^ for *ortho* and *para* positions, respectively. Accordingly, it can be inferred that the position of the methyl substituent on the phenyl ring greatly influences cytotoxicity. Methyl substitution at the *ortho* position, in particular, displayed remarkable cytotoxicity against the tested cancer cell lines. Also, the effect of the nitro functional group on the phenyl-substituted derivatives showed that these electron-poor substations were active at the para position in A-549 cancer cell lines with an IC_50_ value of 16.04 μg mL^−1^. In addition, substitution at the *para* position displayed remarkable cytotoxicity compared to the *meta*-analog substitutions. All these results suggest that the structural features had a profound effect on the biological profile of the compound.

#### 2.2.2 DAPI staining

**Fig. 2:**
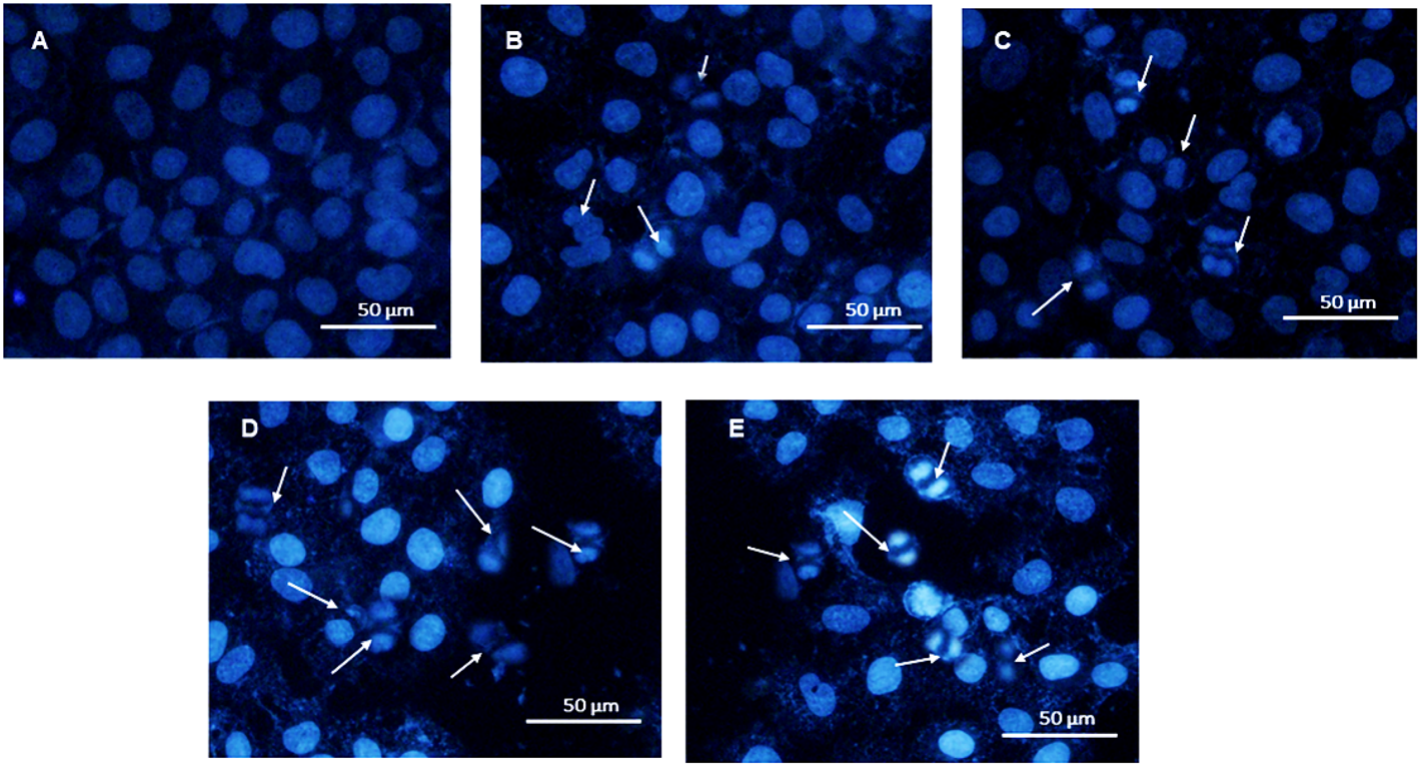
Fluorescent microscope images of non-small-cell lung cancer cell lines A-549 untreated and treated with DHMP derivatives. (A) Control, (B) 5-(1-adamantylcarbamoyl)-6-methyl-4-(4-nitrophenyl)-3,4-dihydropyrimidin-2(1*H*)-thione (IIg), (C) 5-(1-adamantylcarbamoyl)-6-methyl-4-(4-methylphenyl)-3,4-dihydropyrimidin-2(1*H*)-thione (IId), (D) 5-(1-adamantylcarbamoyl)-6-methyl-4-(2-chlorolphenyl)-3,4-dihydropyrimidin-2(1*H*)-thione (IIj), (E) 5-(1-adamantylcarbamoyl)-6-methyl-4-(2-methylphenyl)-3,4-dihydropyrimidin-2(1*H*)-thione (IIb).

DAPI is a dominant chromosome counter stain that binds immutably to the AT clusters in the minor groove of DNA, revealing the nuclear contraction through chromatin condensation. DAPI penetrates less efficiently into the flawless membrane in comparison to the disintegrated membrane, thereby determining the apoptotic cells from the live ones. Hence, apoptotic cells appear brighter than live cells. Therefore, it is critical to study the nuclear damage induced by IIb, IIj, IId, and IIg compounds in A-549 by DAPI staining.^16^ As shown in Fig. 2, the living cells in the control group (A) showed normal morphology and the cell nuclei remained intact. Although A-549 cells were treated with samples for 48 h, the cells exhibited apoptotic features, including nuclear shrinkage, pyknotic or fragmented bright nuclei, and chromatin condensation.

#### 2.2.3 Apoptotic study

**Fig. 3:**
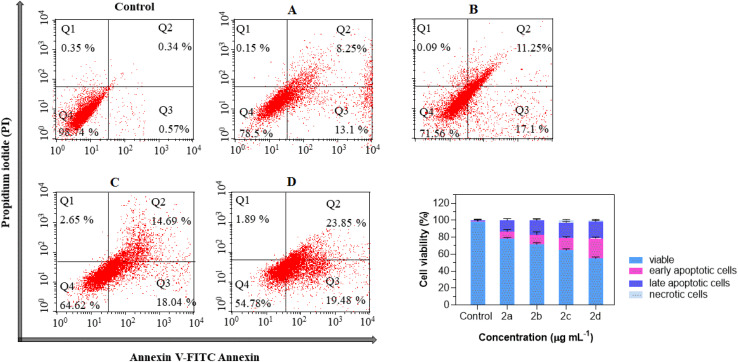
Flow cytometric analysis of non-small-cell lung cancer cell lines A-549 apoptosis induced by different treatment groups for 48 h by using Annexin V/PI staining. Data are shown as mean ± SD, *n* = 3 in all cases. Control, (A) 5-(1-adamantylcarbamoyl)-6-methyl-4-(4-nitrophenyl)-3,4-dihydropyrimidin-2(1*H*)-thione (IIg), (B) 5-(1-adamantylcarbamoyl)-6-methyl-4-(4-methylphenyl)-3,4-dihydropyrimidin-2(1*H*)-thione (IId), (C) 5-(1-adamantylcarbamoyl)-6-methyl-4-(2-chlorolphenyl)-3,4-dihydropyrimidin-2(1*H*)-thione (IIj), (D) 5-(1-adamantylcarbamoyl)-6-methyl-4-(2-methylphenyl)-3,4-dihydropyrimidin-2(1*H*)-thione (IIb).

Annexin V-FITC/propidium iodide dual staining assay expedites the quantification of apoptotic cell death using flow cytometry. Fig. 3 shows the findings of Annexin/PI staining related to identifying living, apoptotic, late apoptotic, and dead A-549 cancer cell lines. We found that the DHPM derivatives IIb, IIj, IId, and IIg increased the overall percentage of apoptosis compared to control groups, with total apoptosis ranging from 21.5% to 45.22%. Also, the 5-(1-adamantylcarbamoyl)-6-methyl-4-(2-methylphenyl)-3,4-dihydropyrimidin-2(1*H*)-thione (IIb) had the highest activity (Fig. 3).

Overall, the selected DHPM derivatives treated with A-549 cancer cell lines increased their sensitivity to apoptosis, with varying percentages in different phases, thereby boosting the overall apoptosis rate. Recent studies have shown that a series of substituted dihydropyrimidinone treatment groups effectively induced apoptosis in various cancer cell lines.^17^

## 3. Experimental section

### 3.1 Chemistry

#### 3.1.1 General

The ^1^H and ^13^C NMR spectra were recorded using Varian INOVA 500 MHz and Bruker 250 MHz NMR spectrometers. The IR spectra were recorded on a Bruker PS-15 spectrometer. Melting points were measured with an Electrothermal 9100 apparatus using open capillaries, without correction. Elemental analyses were performed on a Carlo-Erba 1104 CHN analyzer. All commercial reagents were used without prior purification.

#### 3.1.2 Typical procedure for the synthesis of dihydropyrimidine derivatives IIa–k

The mixture of *N*-(adamant-1-yl)acetoacetamide (235 mg, 1 mmol), benzaldehyde (0.1 mL, 1 mmol), thiourea (76 mg, 1 mmol) and TFA (1.6 μL, 2 mol%) was stirred under preheated conditions at 50 °C until completion of the reaction as indicated by TLC (60 min). The precipitated solid washed with water and recrystallized from ethanol (EtOH). Compound IIa was obtained as white crystals.

##### 3.1.2.1 5-(1-Adamantylcarbamoyl)-6-methyl-4-phenyl-3,4-dihydropyrimidin-2(1*H*)-thione (IIa)

White solid; yield: 83%; mp 238 °C; IR (KBr): *ῡ* = 3396, 3244, 3173 (NH), 2906, 2848 (C–H), 1619 (C=O), 1559 (C=C), 1331 (C–N), 1203 (C=S) cm^−1^; ^1^H NMR (DMSO-*d*_6_, 500 MHz): *δ* = 1.57 (s br, 6H, Ad, C=C–CH_3_), 1.84 (s, 6H, Ad), 1.94 (s, 6H, Ad), 5.21 (s, 1H, C(4)-H), 7.10 (s, 1H, NH–C=O), 7.20 (d, *J* = 8 Hz, 2H, Ar-H), 7.25 (t, *J* = 7.5 Hz, 1H, Ar-H), 7.33 (t, *J* = 7.5 Hz, 2H, Ar-H), 9.18 (s, 1H, NH), 9.73 (s, 1H, NH) ppm; ^13^C NMR (DMSO-*d*_6_, 125 MHz): *δ* = 16.2 (C=C–CH_3_), 28.8 (3C-Ad), 36.0 (3C-Ad), 40.9 (3C-Ad), 51.2 (C-Ad), 55.4 (CH), 108.5 (=C), 143.2 (=C), 126.3 (C_2_, C_6_), 127.5 (C_4_), 128.4 (C_3_, C_5_), 132.63 (C_1_), 165.5 (C=O), 174.1 (C=S) ppm; anal. calcd for C_22_H_27_N_3_OS: C, 69.26; H, 7.13; N, 11.01. Found: C, 69.29; H, 7.27; N, 10.98.

##### 3.1.2.2 5-(1-Adamantylcarbamoyl)-6-methyl-4-(2-methylphenyl)-3,4-dihydropyrimidin-2(1*H*)-thione (IIb)

White solid; yield: 85%; mp 258 °C; IR (KBr): *ῡ* = 3382, 3244, 3187 (NH), 2907, 2845 (C–H), 1627 (C=O), 1597 (C=C), 1332 (C–N), 1197 (C=S) cm^−1^; ^1^H NMR (DMSO-*d*_6_, 500 MHz): *δ* = 1.51 (s, 3H, C=C–CH_3_), 1.54 (s, 3H, Ad), 1.75 (s, 6H, Ad), 1.92 (s, 6H, Ad), 2.34 (s, 3H, Ar-CH_3_), 5.42 (s, 1H, C(4)-H), 7.01 (s, 1H, NH–C=O), 7.11 (t, *J* = 7.5 Hz, 1H, Ar-H), 7.15 (d, *J* = 7 Hz, 1H, Ar-H), 7.18 (d, *J* = 7.5 Hz, 1H, Ar-H), 7.21 (t, *J* = 7.5 Hz, 1H, Ar-H), 8.99 (s, 1H, NH), 9.66 (s, 1H, NH) ppm; ^13^C NMR (DMSO-*d*_6_, 125 MHz): *δ* = 16.0 (C=C–CH_3_), 18.9 (Ar-CH_3_), 28.7 (3C-Ad), 36.0 (3C-Ad), 40.8 (3C-Ad), 51.0 (C-Ad), 52.7 (C(4)-H), 108.5 (=C), 141.6 (=C), 126.3 (C_5_), 127.4 (C_3_), 128.3 (C_4_), 130.1 (C_6_), 132.0 (C_2_), 134.9 (C_1_-phenyl), 165.4 (C=O), 173.3 (C=S) ppm; anal. calcd for C_23_H_29_N_3_OS: C, 69.84; H, 7.39; N, 10.62. Found: C, 69.78; H, 7.09; N, 10.88.

##### 3.1.2.3 5-(1-Adamantylcarbamoyl)-6-methyl-4-(3-methylphenyl)-3,4-dihydropyrimidin-2(1*H*)-thione (IIc)

White solid; yield: 81%; mp 256 °C; IR (KBr): *ῡ* = 3400, 3195, 3088 (NH), 2905, 2851 (C–H), 1617 (C=O), 1559 (C=C), 1197 (C=S) cm^−1^; ^1^H NMR (DMSO-*d*_6,_ 500 MHz): *δ* = 1.57 (s, 6H, Ad), 1.85 (s, 6H, Ad), 1.92 (s, 3H, C=C–CH_3_), 1.96 (s, 3H, Ad), 2.28 (s, 3H, Ar-CH_3_), 5.18 (s, 1H, C(4)-H), 6.98–7.00 (m, 2H, Ar-H), 7.07 (d, *J* = 7.5 Hz, 1H, Ar-H), 7.10 (s, 1H, NH–C=O), 7.22 (t, *J* = 9 Hz, 1H, Ar-H), 9.13 (s, 1H, NH), 9.69 (s, 1H, NH) ppm; ^13^C NMR (DMSO-*d*_6,_ 125 MHz): *δ* = 16.2 (C=C–CH_3_), 21.15 (Ar-CH_3_), 28.8 (3C-Ad), 36.0 (3C-Ad), 40.9 (3C-Ad), 51.2 (C-Ad), 55.3 (CH), 108.6 (=C), 143.1 (=C), 123.3 (C_6_), 126.8 (C_4_), 127.9 (C_2_), 128.3 (C_5_), 132.3 (C_3_), 137.3 (C_1_), 165.5 (C=O), 174.1 (C=S) ppm; anal. calcd for C_23_H_29_N_3_OS: C, 69.84; H, 7.39; N, 10.62. Found: C, 69.98; H, 7.19; N, 10.78.

##### 3.1.2.4 5-(1-Adamantylcarbamoyl)-6-methyl-4-(4-methylphenyl)-3,4-dihydropyrimidin-2(1*H*)-thione (IId)

White solid; yield: 70%; mp 230 °C; IR (KBr): *ῡ* = 3411, 3241, 3187 (NH), 2917, 2850 (C–H), 1616 (C=O), 1555 (C=C), 1385 (C–N), 1201 (C=S) cm^−1^; ^1^H NMR (DMSO-*d*_6,_ 500 MHz): *δ* = 1.57 (s, 6H, Ad), 1.85 (s, 6H, Ad), 1.93 (s, 3H, C=C–CH_3_), 1.96 (s, 3H, Ad), 2.26 (s, 3H, Ar-CH_3_), 5.16 (s, 1H, C(4)-H), 7.09–7.14 (m, 4H, Ar-H), 7.09 (s, 1H, NH–C=O), 9.14 (s, 1H, NH), 9.69 (s, 1H, NH) ppm; ^13^C NMR (DMSO-*d*_6,_ 125 MHz): *δ* = 16.2 (C=C–CH_3_), 20.6 (Ar-CH_3_), 28.8 (3C-Ad), 36.0 (3C-Ad), 40.9 (3C-Ad), 51.2 (C-Ad), 55.0 (CH), 108.6 (=C), 140.3 (=C), 126.2 (C_2_, C_6_), 128.9 (C_3_, C_5_), 132.5 (C_4_), 136.5 (C_1_), 165.5 (C=O), 173.9 (C=S) ppm; anal. calcd for C_23_H_29_N_3_OS: C, 69.84; H, 7.39; N, 10.62. Found: C, 69.68; H, 7.59; N, 10.98.

##### 3.1.2.5 5-(1-Adamantylcarbamoyl)-6-methyl-4-(3-methoxyphenyl)-3,4-dihydropyrimidin-2(1*H*)-thione (IIe)

White solid; yield: 74%; mp 256–259 °C; IR (KBr): *ῡ* = 3392, 3180, 3085 (NH), 2901, 2850 (C–H), 1615 (C=O), 1557 (C=C), 1198 (C=S) cm^−1^; ^1^H NMR (DMSO-*d*_6,_ 500 MHz): *δ* = 1.60 (s, 6H, Ad), 1.89 (s, 6H, Ad), 1.96 (s, 3H, C=C–CH_3_), 1.99 (s, 3H, Ad), 3.75 (s, 3H, Ar-OCH_3_), 5.21 (s, 1H, C(4)-H), 6.77 (s, 1H, Ar-H), 6.81 (d, *J* = 7.5 Hz, 1H, Ar-H), 6.85 (d, *J* = 8 Hz, 1H, Ar-H), 7.16 (s, 1H, NH–C=O), 7.28 (t, *J* = 7.5 Hz, 1H, Ar-H), 9.19 (s, 1H, NH), 9.76 (s, 1H, NH) ppm; ^13^C NMR (DMSO-*d*_6,_ 125 MHz): *δ* = 16.2 (C=C–CH_3_), 28.8 (3C-Ad), 36.0 (3C-Ad), 40.9 (3C-Ad), 51.2 (C-Ad), 54.9 (CH), 55.1 (Ar-OCH_3_), 108.5 (=C), 159.3 (=C), 112.1 (C_4_), 112.4 (C_2_), 118.3 (C_6_), 129.5 (C_5_), 132.7 (C_1_), 144.7 (C_3_), 165.4 (C=O), 174.2 (C=S) ppm; anal. calcd for C_23_H_29_N_3_O_2_S: C, 67.12; H, 7.10; N, 10.21. Found: C, 67.44; H, 7.03; N, 10.35.

##### 3.1.2.6 13-(1-Adamantylcarbamoyl)-9-methyl-11-thioxo-8-oxa-10,12-diazatricyclo [7.3.1.0^2,7^] trideca-2,4,6-triene (IIf)

Yellow solid; yield: 75%; mp > 250 °C; IR (KBr): *ῡ* = 3352, 3277 (NH), 2914, 2850 (C–H), 1633 (C=O), 1229 (C=S), 1154 (C–O) cm^−1^; ^1^H NMR (DMSO-*d*_6,_ 500 MHz): *δ* = 1.62 (s, 6H, Ad), 1.66 (s, 3H, C–C–CH_3_), 1.92 (s, 6H, Ad), 2.01 (s, 3H, Ad), 2.91 (s, 1H, CH-13), 4.40 (d, *J* = 4 Hz, 1H, CH-1), 6.80 (d, *J* = 8 Hz, 1H, Ar-H), 6.91 (t, *J* = 7.5 Hz, 1H, Ar-H), 7.15 (d, *J* = 7.5 Hz, 1H, Ar-H), 7.19 (t, *J* = 7.5 Hz, 1H, Ar-H), 7.54 (s, 1H, NH–C=O), 8.86 (s, 1H, NH), 8.93 (d, *J* = 4 Hz, 1H, NH) ppm; ^13^C NMR (DMSO-*d*_6,_ 125 MHz): *δ* = 22.8 (CH_3_), 28.8 (3C-Ad), 36.0 (3C-Ad), 40.8 (3C-Ad), 42.0 (CH-13), 49.1 (C-Ad), 50.9 (CH-1), 81.8 (C-9), 116.4 (C_3_), 120.5 (C_5_), 124.9 (C_4_), 128.6 (C_6_), 129.4 (C_2_), 150.7 (C_7_), 166.6 (C=O), 176.1 (C=S) ppm; anal. calcd for C_22_H_27_N_3_O_2_S: C, 66.47; H, 6.85; N, 10.57. Found: C, 66.87; H, 7.08; N, 10.37.

##### 3.1.2.7 5-(1-Adamantylcarbamoyl)-6-methyl-4-(3-hydroxyphenyl)-3,4-dihydropyrimidin-2(1*H*)-thione (IIh)

White solid; yield: 80%; mp 183–185 °C; IR (KBr): *ῡ* = 3380, 3323, 3217 (NH, OH, broad), 2908, 2852 (C–H), 1619 (C=O), 1197 (C=S) cm^−1^; ^1^H NMR (DMSO-*d*_6,_ 500 MHz): *δ* = 1.57 (s, 6H, Ad), 1.86 (s, 6H, Ad), 1.92 (s, 3H, Ad), 1.98 (s, 3H, CH_3_), 5.12 (s, 1H, C(4)-H), 6.61–6.62 (m, 3H, Ar-H), 7.04 (s, 1H, NH–C=O), 7.10 (t, *J* = 7.5 Hz, 1H, Ar-H), 9.12 (s, O–H), 9.38 (s, 1H, NH), 9.68 (s, 1H, NH) ppm; ^13^C NMR (DMSO-*d*_6,_ 125 MHz): *δ* = 16.2 (C=C–CH_3_), 28.8 (3C-Ad), 36.1 (3C-Ad), 40.9 (3C-Ad), 51.2 (C-Ad), 55.4 (CH), 108.6 (=C), 157.5 (=C), 113.3 (C_4_), 114.4 (C_2_), 116.9 (C_6_), 129.2 (C_5_), 132.5 (C_1_), 144.7 (C_3_), 165.5 (C=O), 174.0 (C=S) ppm; anal. calcd for C_22_H_27_N_3_O_2_S: C, 66.47; H, 6.85; N, 10.57. Found: C, 66.69; H, 7.12; N, 10.37.

##### 3.1.2.8 5-(1-Adamantylcarbamoyl)-6-methyl-4-(3-nithrophenyl)-3,4-dihydropyrimidin-2(1*H*)-thione (IIi)

Yellow solid; yield: 60%; mp > 270 °C; IR (KBr): *ῡ* = 3373, 3163 (br. s, NH), 2906, 2848 (C–H), 1642 (C=O), 1566 (C=C), 1529, 1331 (NO_2_), 1202 (C=S) cm^−1^; ^1^H NMR (DMSO-*d*_6,_ 500 MHz): *δ* = 1.56 (s br, 6H, Ad, C=C–CH_3_), 1.83 (s, 6H, Ad), 1.95 (s, 6H, Ad), 5.36 (s, 1H, C(4)-H), 7.27 (s, 1H, Ar-H), 7.64–7.69 (m, 2H, Ar-H), 8.06 (s, 1H, NH–C=O), 8.15 (d, *J* = 8 Hz, 1H, Ar-H), 9.34 (s, 1H, NH), 9.93 (s, 1H, NH) ppm; ^13^C NMR (DMSO-*d*_6,_ 62.5 MHz): *δ* = 16.0 (C=C–CH_3_), 28.7 (3C-Ad), 36.0 (3C-Ad), 40.8 (3C-Ad), 51.3 (C-Ad), 54.8 (CH), 107.5 (=C), 147.8 (=C), 121.0 (C_5_), 121.5 (C_4_), 122.5 (C_2_), 130.1 (C_6_), 133.5 (C_1_), 145.1 (C_3_), 165.2 (C=O), 174.4 (C=S) ppm; anal. calcd for C_22_H_26_N_4_O_3_S: C, 61.95; H, 6.14; N, 13.14. Found: C, 61.69; H, 6.46; N, 13.18.

##### 3.1.2.9 5-(1-Adamantylcarbamoyl)-6-methyl-4-(4-nithrophenyl)-3,4-dihydropyrimidin-2(1*H*)-thione (IIg)

Yellow solid; yield: 68%; mp 216–218 °C; IR (KBr): *ῡ* = 3369, 3185 (NH), 2912, 2853 (CH), 1619 (C=O), 1530, 1346 (NO_2_), 1250 (C=S) cm^−1^; ^1^H NMR (DMSO-*d*_6_, 250 MHz): *δ* = 1.55 (s br, 6H, Ad, C=C–CH_3_), 1.83 (s, 6H, Ad), 1.93 (s, 6H, Ad), 5.29 (s, 1H, C(4)-H), 7.23 (s, 1H, NH–C=O), 7.43 (d, *J* = 8 Hz, 2H, Ar-H), 8.22 (d, *J* = 8 Hz, 2H, Ar-H), 9.31 (s, 1H, NH), 9.89 (s, 1H, NH) ppm; ^13^C NMR (DMSO-*d*_6_, 62.5 MHz):16.75 (C=C–CH_3_), 29.20, 36.42, 41.25, 51.76 (10C-Ad), 55.39 (CH), 107.81, 150.59 (=C), 124.18 (C_2_, C_6_, Ar), 128.09 (C_3_, C_5_, Ar), 133.99 (C_4_), 147.27 (C_1_, Ar), 165.64 (C=O), 174.81 (C=S) ppm; anal. calcd for C_22_H_26_N_4_O_3_S: C, 61.95; H, 6.14; N, 13.14. Found: C, 61.89; H, 6.23; N, 13.33.

##### 3.1.2.10 5-(1-Adamantylcarbamoyl)-6-methyl-4-(2-chlorophenyl)-3,4-dihydropyrimidin-2(1*H*)-thione (IIj)

White solid; yield: 67%; mp 184–186 °C; IR (KBr): *ῡ* = 3405, 3297, 3185 (NH), 2906, 2847 (C–H), 1618 (C=O), 1566 (C=C), 1362 (C–N), 1260 (C=S), 750 (C–Cl) cm^−1^; ^1^H NMR (DMSO-*d*_6,_ 500 MHz): *δ* = 1.51 (s, 6H, Ad), 1.75 (s, 3H, C=C–CH_3_), 1.79 (s, 3H, Ad), 1.91 (s, 6H, Ad), 5.60 (s, 1H, C(4)-H), 7.16 (s, 1H, NH–C=O), 7.26–7.29 (m, 1H, Ar-H), 7.34–7.38 (m, 3H, Ar-H), 9.07 (s, 1H, NH), 9.78 (s, 1H, NH) ppm; ^13^C NMR (DMSO-*d*_6,_ 125 MHz): *δ* = 15.9 (C=C–CH_3_), 28.7 (3C-Ad), 36.0 (3C-Ad), 40.7 (3C-Ad), 51.0 (C-Ad), 53.0 (CH), 107.8 (=C), 140.3 (=C), 127.5 (C_6_), 129.2 (C_3_, C_5_), 129.8 (C_2_), 131.4 (C_4_), 132.6 (C_1_), 164.9 (C=O), 173.9 (C=S) ppm; anal. calcd for C_22_H_26_N_3_ClOS: C, 63.52; H, 6.30; N, 10.10. Found: C, 67.29; H, 6.13; N, 10.40.

##### 3.1.2.11 5-(1-Adamantylcarbamoyl)-6-methyl-4-(3-chlorophenyl)-3,4-dihydropyrimidin-2(1*H*)-thione (IIk)

White solid; yield: 50%; mp 238 °C; IR (KBr) *ῡ* = 3394, 3238, 3174 (NH), 2912, 2849 (CH), 1619 (C=O), 1559 (C=C), 1203 (C=S) cm^−1^; ^1^H NMR (DMSO-*d*_6_, 250 MHz): *δ* = 1.55 (s br, 6H, Ad, C=C–CH_3_), 1.83 (s, 6H, Ad), 1.91 (s, 6H, Ad), 5.197 (s, 1H, C(4)-H), 7.19 (s, 1H, NH), 7.12–7.36 (m, 4H, Ar-H), 9.204 (s, 1H, NH), 9.805 (s, 1H, NH) ppm; ^13^C NMR (DMSO-*d*_6_, 62.5 MHz): *δ* = 16.73 (C=C–CH_3_), 29.20, 36.45, 40, 51.73 (Ad), 55.32 (CH), 108.36, 159 (C=C), 125.41 (C_2,_ Ar),126.64 (C_6_, Ar), 127.82 (C_4_, Ar), 130.82 (C_5_, Ar), 133.45 (C_3_, Ar), 145.86 (C_1_, Ar), 165.81 (C=O), 174.67 (C=S) ppm; anal. calcd for C_22_H_26_N_3_ClOS: C, 63.52; H, 6.30; N, 10.10. Found: C, 67.19; H, 6.36; N, 10.24.

### 3.2 Biological activity of IIa–k

RPMI-1640 medium, penicillin, streptomycin, fetal calf serum, sodium bicarbonate, phosphate-buffered saline, trypsin, gentamycin sulfate, 3-(4,5-dimethylthiazol-2-yl)-2,5-diphenyltetrazolium bromide (MTT), paraformaldehyde, and 4,6-diamidino-2-phenylindole (DAPI) were obtained from Sigma Chemicals Co. Cells undergoing early and late apoptosis were easily distinguished using the eBioscience Annexin V Apoptosis Detection Kit (Annexin V-FITC kit, eBioscience, Vienna, Austria).

#### 3.2.1 *In vitro* cytotoxicity assay

Human adenocarcinoma A-549 cell line was obtained from the Iranian Cell Bank (Pasteur Institute, Iran) and grown in RPMI-1640 medium supplemented 10% FBS at 37 °C and 5% CO_2_. The medium was replaced on alternate days, and cells were trypsinized after reaching confluency. The cytotoxicity of IIa–k compounds was evaluated by MTT assay.^18^ Cells were seeded in 96-well plates at a density of 1 × 10^4^ cells per well and incubated at 37 °C for 24 h. Then, the cells were treated with different concentrations of the prepared DHPM derivatives and incubated for 48 h at 37 °C. Afterward, the medium was discarded, and the cells were incubated with 100 μL of MTT solution at 37 °C for 4 h. The medium was removed completely, and 150 μL of dimethyl sulfoxide (DMSO) was included in each well to dissolve the formazan crystals. At the end of the MTT assay, the absorbance of the resulting solutions was assessed at 570 nm using an ELISA plate reader (Stat Fax 3200; Awareness Technology Inc). Data were expressed as mean ± SD (*n* = 3).

#### 3.2.2 DAPI nucleic acid staining

Morphological variations in the nucleus were detected by DAPI staining.^18,19^ After treatment with IIa–k compounds for 48 h, the lung cancer cell line A-549 cells were washed with phosphate-buffered saline (PBS) and permeabilized with 0.1% Triton X for 10 min followed by staining with 1 μM DAPI. Control and treated cells were examined with a fluorescence microscope with excitation at 359 nm and emission at 461 nm using a DAPI filter at 200× magnification.

#### 3.2.3 Analysis of cellular apoptosis

First, the cells were treated with the prepared IIa–k compounds. After 24 hours of incubation, the cells (1–5 × 10^5^) were collected and suspended in 1× Binding Buffer (500 μL), Annexin V-FITC (5 μL), and PI (5 μL). Finally, cells were incubated for 5 min in the dark at 37 °C. Annexin V-FITC conjugate is particularly formulated to provide in improved fluorescence signal and photostability. The Annexin V-FITC kit contains Annexin V-FITC and PI for detecting apoptosis and necrosis, respectively. Necrosis and apoptosis can be characterized in this manner. The detection and analysis of Annexin V-FITC binding were performed *via* flow cytometry, with excitation at 488 nm and emission at 530 nm. FITC signal detection, typically recorded in FL1, was employed for Annexin V binding assessment. Concurrently, propidium iodide (PI) staining was carried out, and the phycoerythrin emission signal, typically recorded in FL2, was utilized for PI detection.^19,20^

#### 3.2.4 Statistical analysis

Statistical analysis was carried out by GraphPad Prism 8.0. The results were reported using the one-way Analysis of Variance (ANOVA). All analyses were completed in triplicates and expressed as mean ± SD (*n* = 3). A *P*-value <0.05 was considered as statistically significant.

## 4. Conclusion

In this study, we developed a solvent-free method for synthesizing DHPMs using TFA as an efficient catalyst for the Biginelli-type reaction. This method enables the one-pot, three-component condensation of *N*-(adamant-1-yl)acetoacetamide, benzaldehyde derivatives, and thiourea. The technique improves upon the original Biginelli reaction by enhancing yields and reducing reaction times under solvent-free conditions. A diverse library of DHPM analogs was synthesized using this approach, and many derivatives exhibited significant anti-proliferative activity against A-549 lung cancer cells. Notably, compounds IIb, IIj, IId, and IIg demonstrated particularly high cytotoxic activity. As can be seen, the compound 5-(1-adamantylcarbamoyl)-6-methyl-4-(2-methylphenyl)-3,4-dihydropyrimidin-2(1*H*)-thione (IIb) showed even better cytotoxicity manner than the common anticancer drug (doxorubicin). Further analysis with DAPI staining and Annexin V-FITC apoptosis assays revealed that these compounds induced apoptosis in A-549 cells. Overall, the synthesized DHPM derivatives show promising potential for development into novel cancer therapeutics with appropriate structural modifications.

## Data availability

The data supporting the findings of this study are included in the ESI† file. Additional data will be available upon the request.

## Conflicts of interest

The authors declare that they have no known competing financial interests or personal relationships that could have appeared to influence the work reported in this paper.

## Supplementary Material

RA-015-D5RA00284B-s001
