# Supplementary material for: Green synthesis of new adamantane-containing dihydropyrimidine derivatives as potential anticancer agents
Source: RSC Adv. 2025 Mar 13;15(10):7949–55. doi: 10.1039/d5ra00284b (PMC11904882; doi:10.1039/d5ra00284b)

Supplementary Information (SI) for RSC Advances.
This journal is © The Royal Society of Chemistry 2025

Green synthesis of new adamantane-containing dihydropyrimidine derivatives as potential anticancer agents

Adeleh Moshtaghi Zonouz[a*], Mina Abkar Aras[a], Nahideh Jafari[b], Zahra Rezaei[a], Hamed Hamishehkar[c*]

[a]*Department of Chemistry, Faculty of Science, Azarbaijan Shahid Madani University, Tabriz- Iran.*

[b] *Faculty of Chemical and Petroleum Engineering, University of Tabriz, Tabriz, Iran*

[c]*Drug Applied Research Center, Tabriz University of Medical Sciences, Tabriz, Iran.*

Corresponding authors:

Adeleh Moshtaghi Zonouz: adelehmz@yahoo.com.

Hamed Hamishehkar: hamishehkarh@tbzmed.ac.ir; Hamishehkar.hamed@gmail.com.

FTIR 5-(1-adamantylcarbamoyl) −6-methyl-4-phenyl-3,4-dihydropyrimidin-2(1H)-thione [IIa]

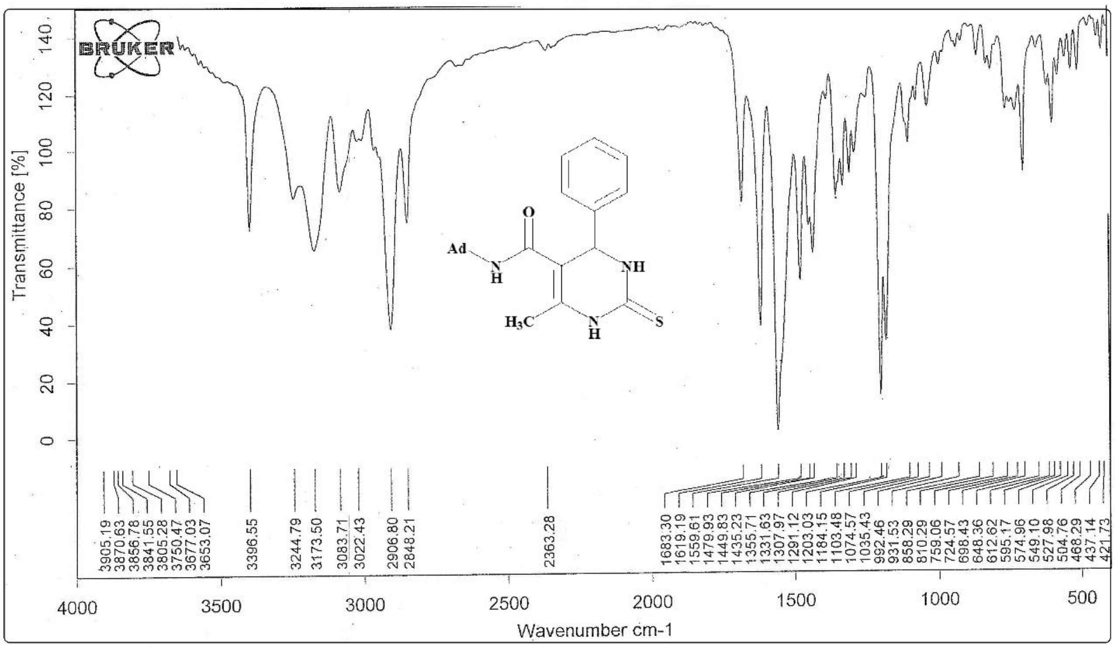

^{1}HNMR of 5-(1-adamantylcarbamoyl) −6-methyl-4-phenyl-3,4-dihydropyrimidin-2(1H)-thione [IIa]

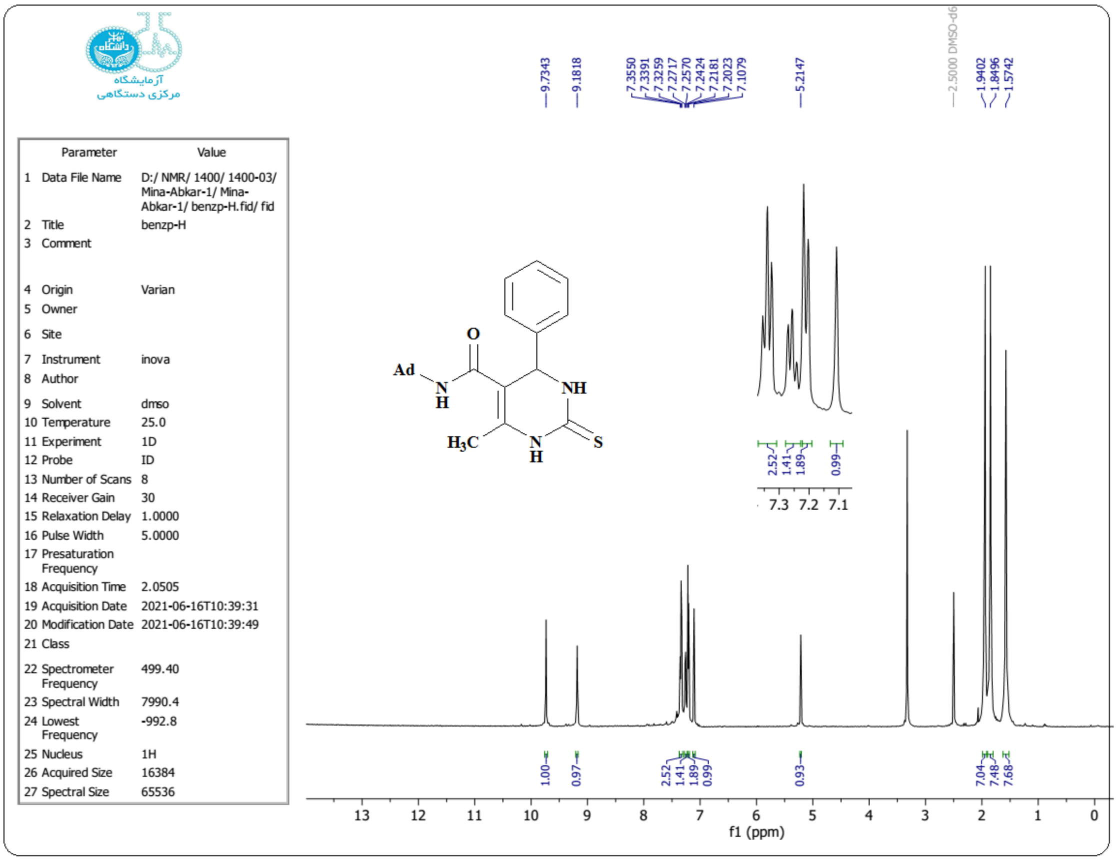

^{13}CNMR of 5-(1-adamantylcarbamoyl) −6-methyl-4-phenyl-3,4-dihydropyrimidin-2(1H)-thione [IIa]

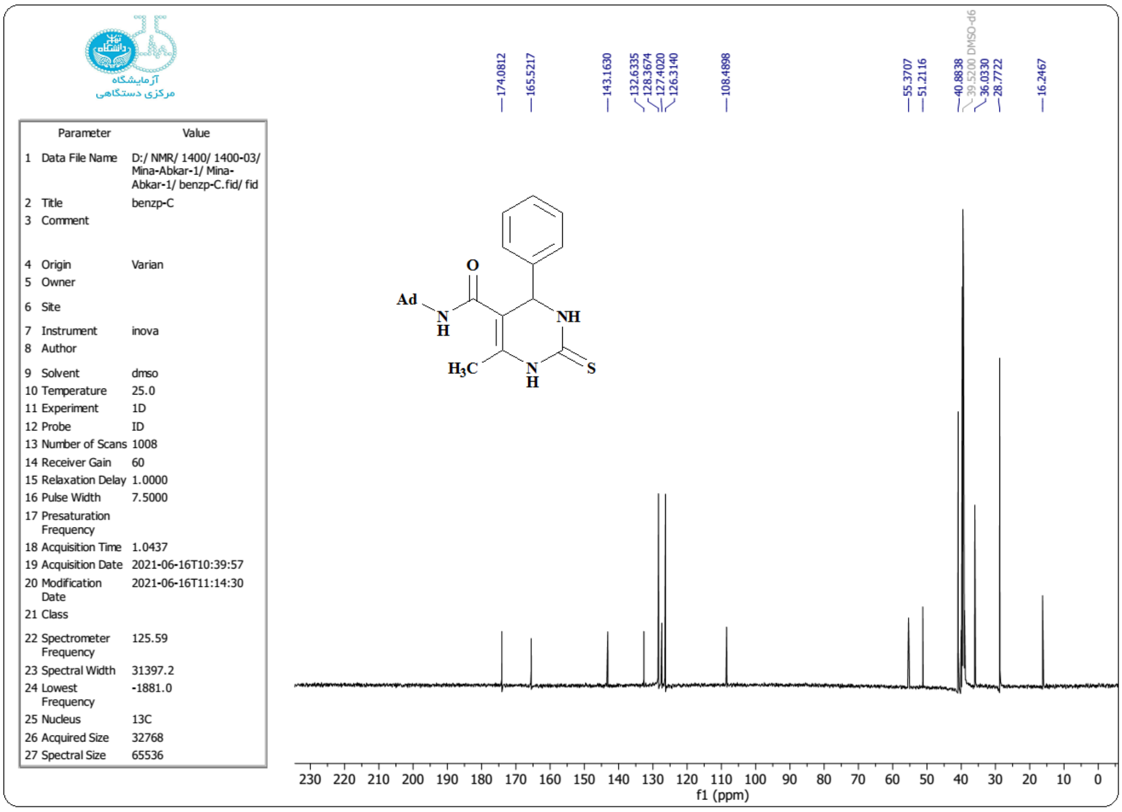

FTIR of 5-(1-adamantylcarbamoyl) −6-methyl-4-(2-methylphenyl) −3,4-dihydropyrimidin-2(1H)-thione [IIb]

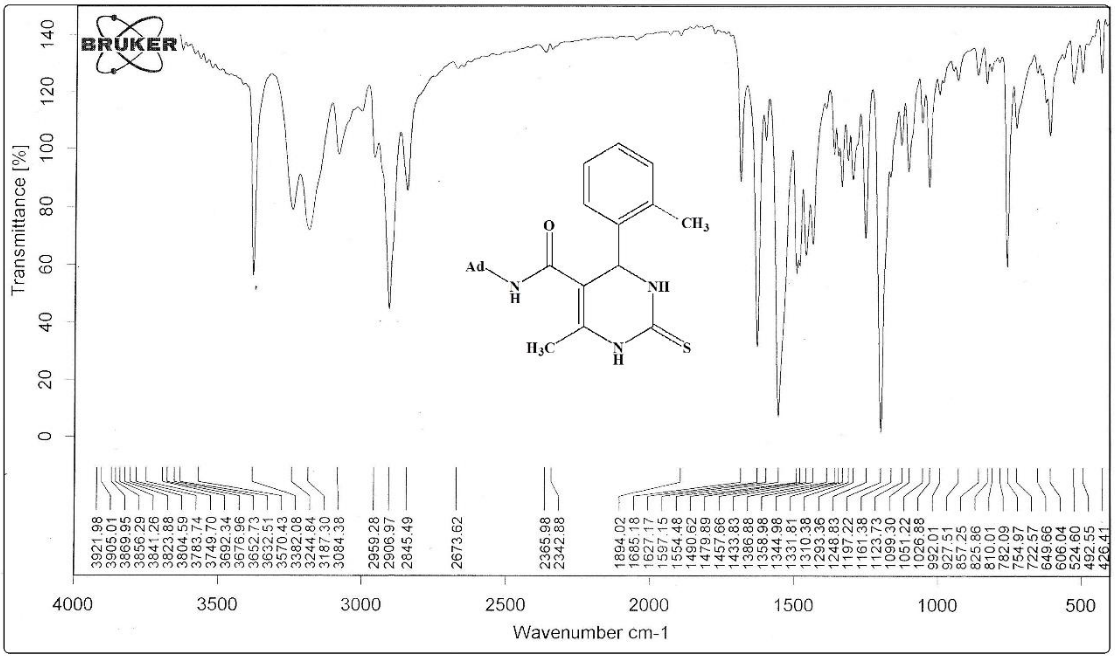

^{1}HNMR of 5-(1-adamantylcarbamoyl) −6-methyl-4-(2-methylphenyl) −3,4-dihydropyrimidin-2(1H)-thione [IIb]

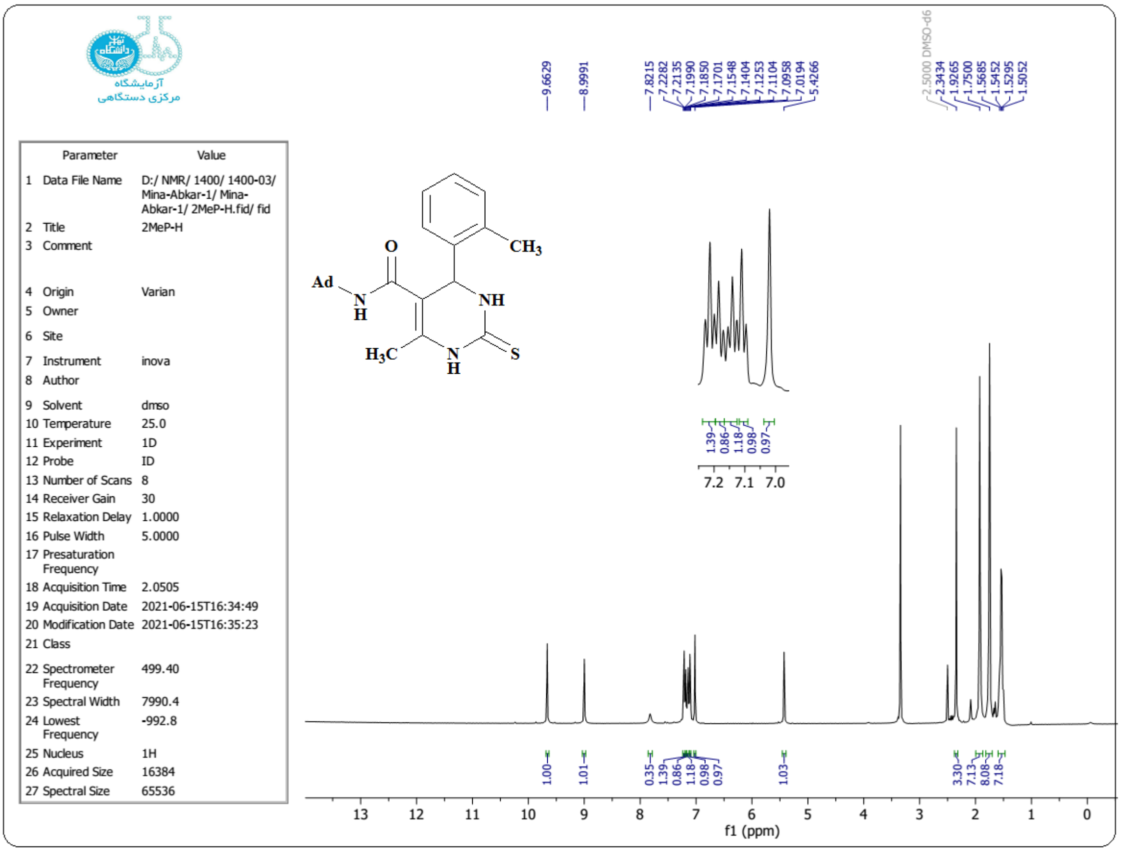

^{13}CNMR of 5-(1-adamantylcarbamoyl) −6-methyl-4-(2-methylphenyl) −3,4-dihydropyrimidin-2(1H)-thione [IIb]

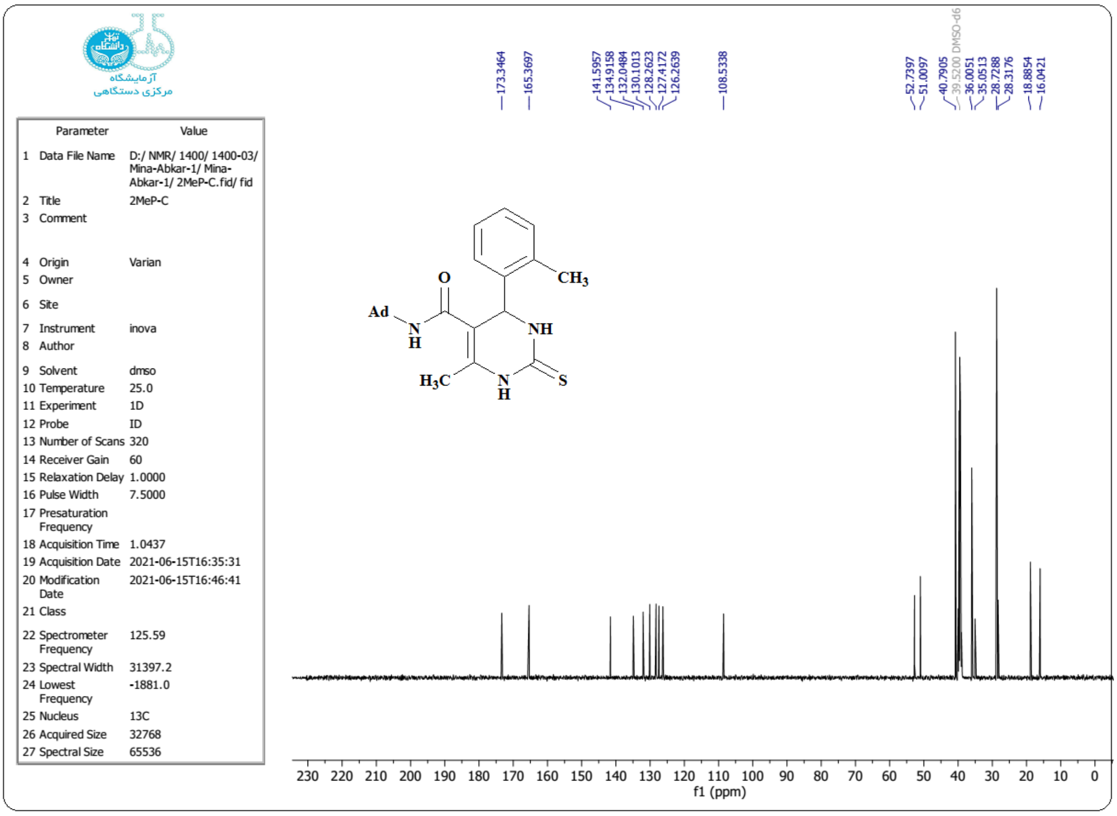

FTIR of 5-(1-adamantylcarbamoyl) −6-methyl-4-(3-methylphenyl) −3,4-dihydropyrimidin-2(1H)-thione [IIc]

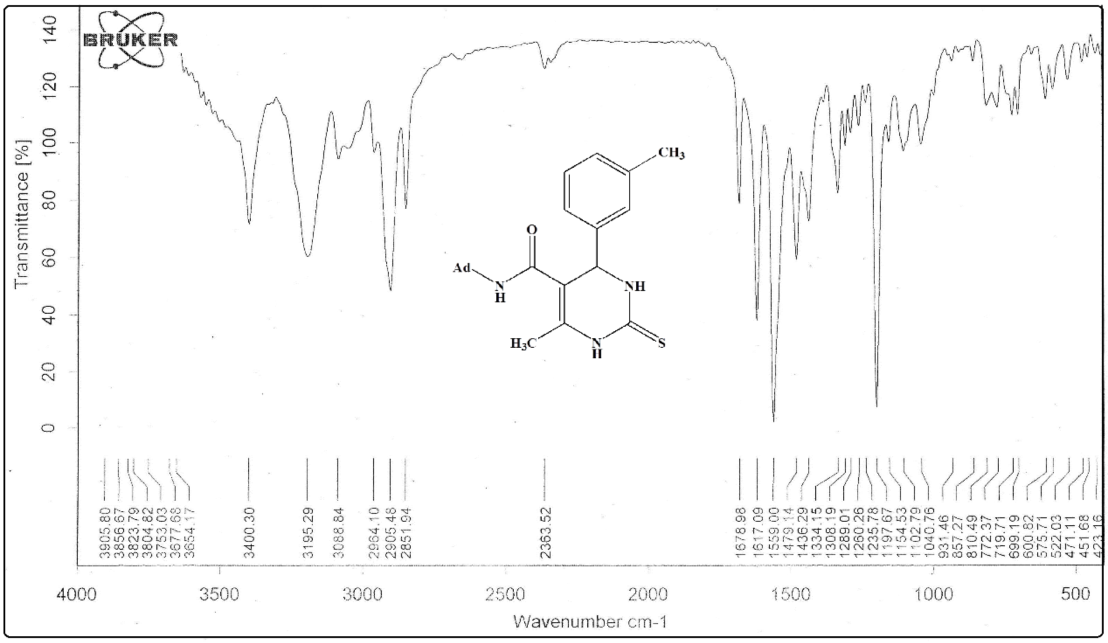

^{1}HNMR of 5-(1-adamantylcarbamoyl) −6-methyl-4-(3-methylphenyl) −3,4-dihydropyrimidin-2(1H)-thione [IIc]

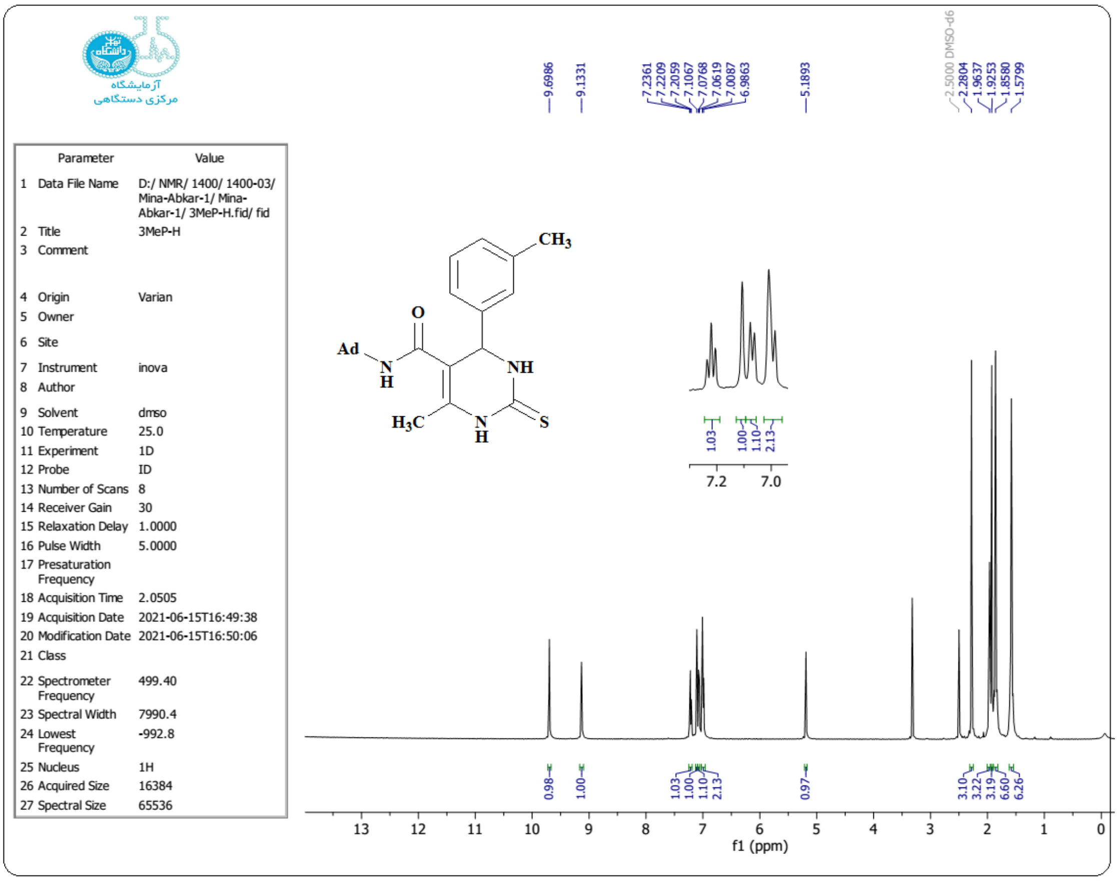

	Parameter	Value
1	Data File Name	D:/ NMR/ 1400/ 1400-03/ Mina-Abkar-1/ Mina-Abkar-1/ 3MeP-H.fid/ fid
2	Title	3MeP-H
3	Comment	
4	Origin	Varian
5	Owner	
6	Site	
7	Instrument	inova
8	Author	
9	Solvent	dmso
10	Temperature	25.0
11	Experiment	1D
12	Probe	ID
13	Number of Scans	8
14	Receiver Gain	30
15	Relaxation Delay	1.0000
16	Pulse Width	5.0000
17	Presaturation Frequency	
18	Acquisition Time	2.0505
19	Acquisition Date	2021-06-15T16:49:38
20	Modification Date	2021-06-15T16:50:06
21	Class	
22	Spectrometer Frequency	499.40
23	Spectral Width	7990.4
24	Lowest Frequency	-992.8
25	Nucleus	1H
26	Acquired Size	16384
27	Spectral Size	65536

^{13}CNMR of 5-(1-adamantylcarbamoyl) −6-methyl-4-(3-methylphenyl) −3,4-dihydropyrimidin-2(1H)-thione [IIc]

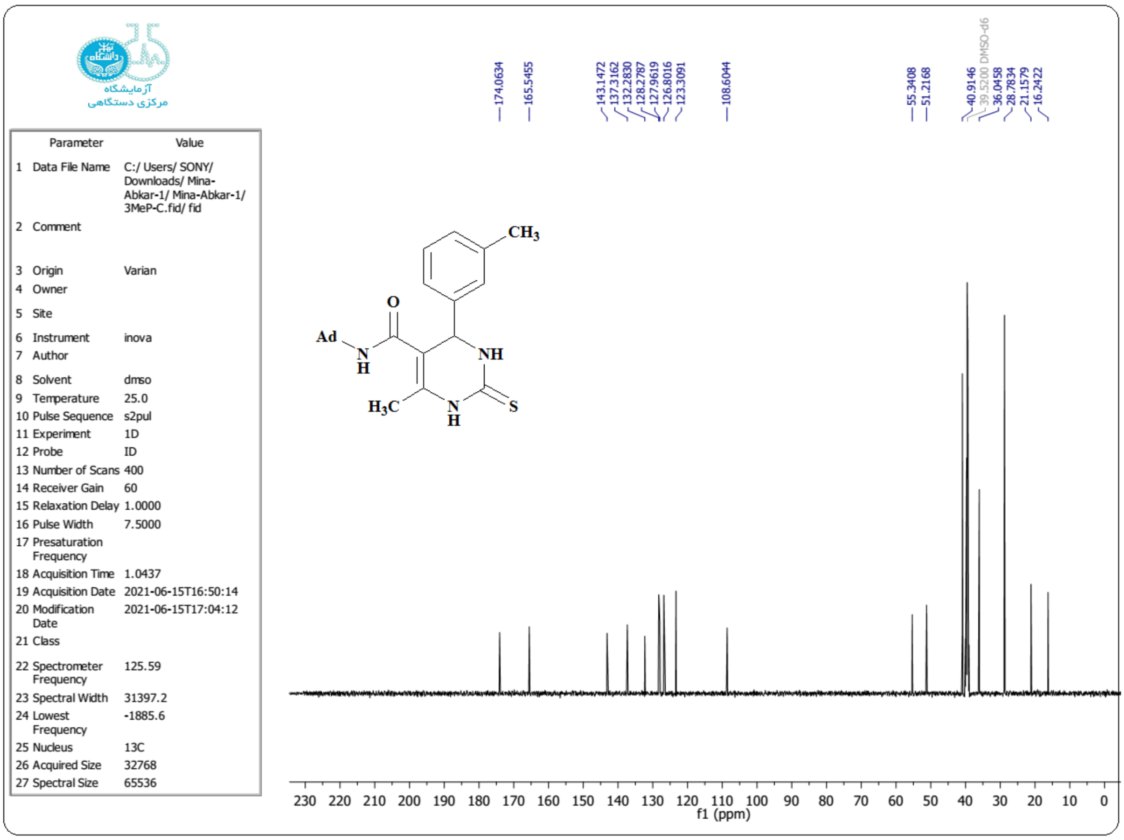

FTIR of 5-(1-adamantylcarbamoyl) −6-methyl-4-(4-methylphenyl) −3,4-dihydropyrimidin-2(1H)-thione [IId]

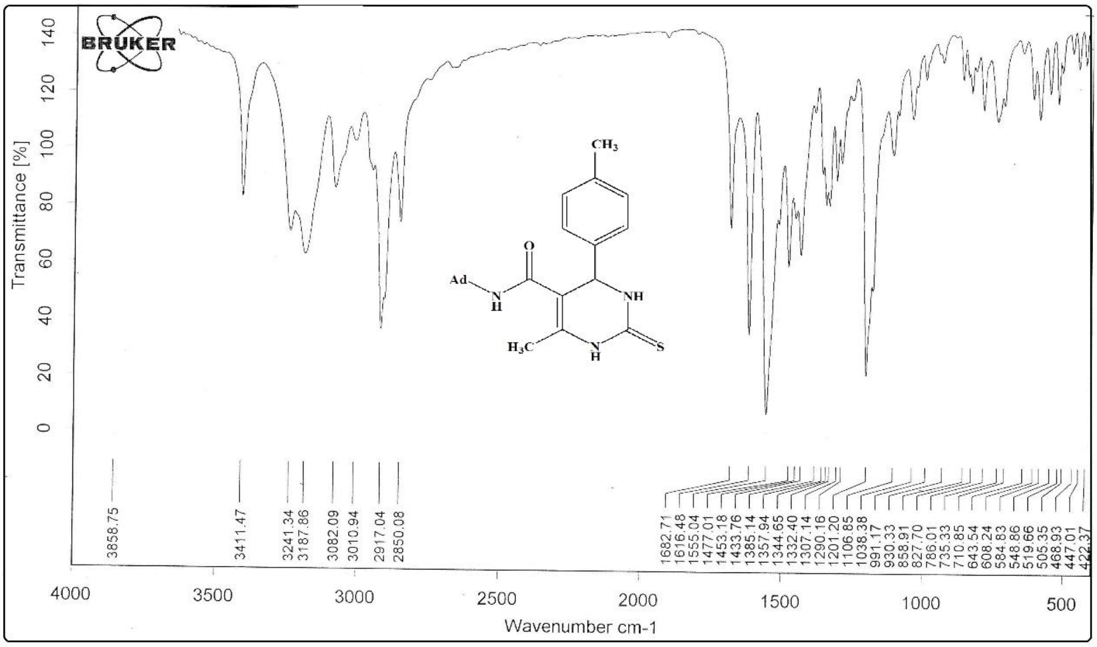

^{1}HNMR of 5-(1-adamantylcarbamoyl) −6-methyl-4-(4-methylphenyl) −3,4-dihydropyrimidin-2(1H)-thione [IId]

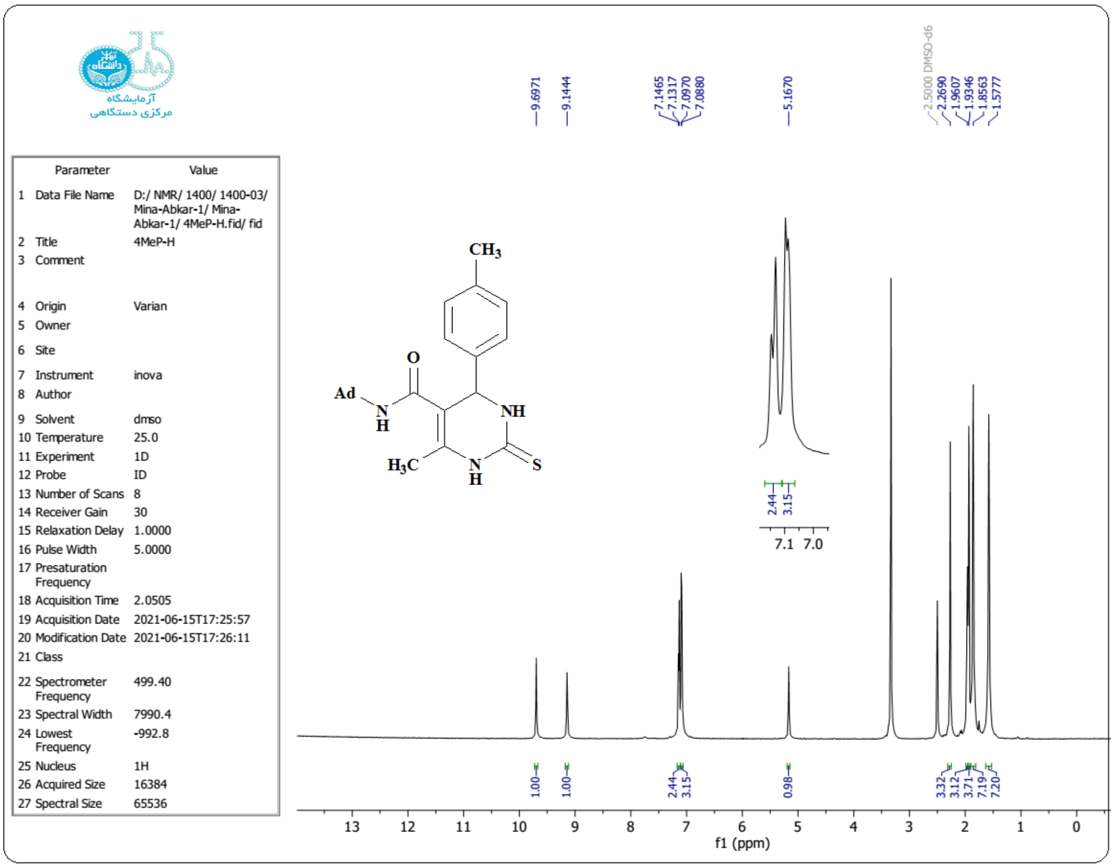

^{13}CNMR of 5-(1-adamantylcarbamoyl) −6-methyl-4-(4-methylphenyl) −3,4-dihydropyrimidin-2(1H)-thione [IId]

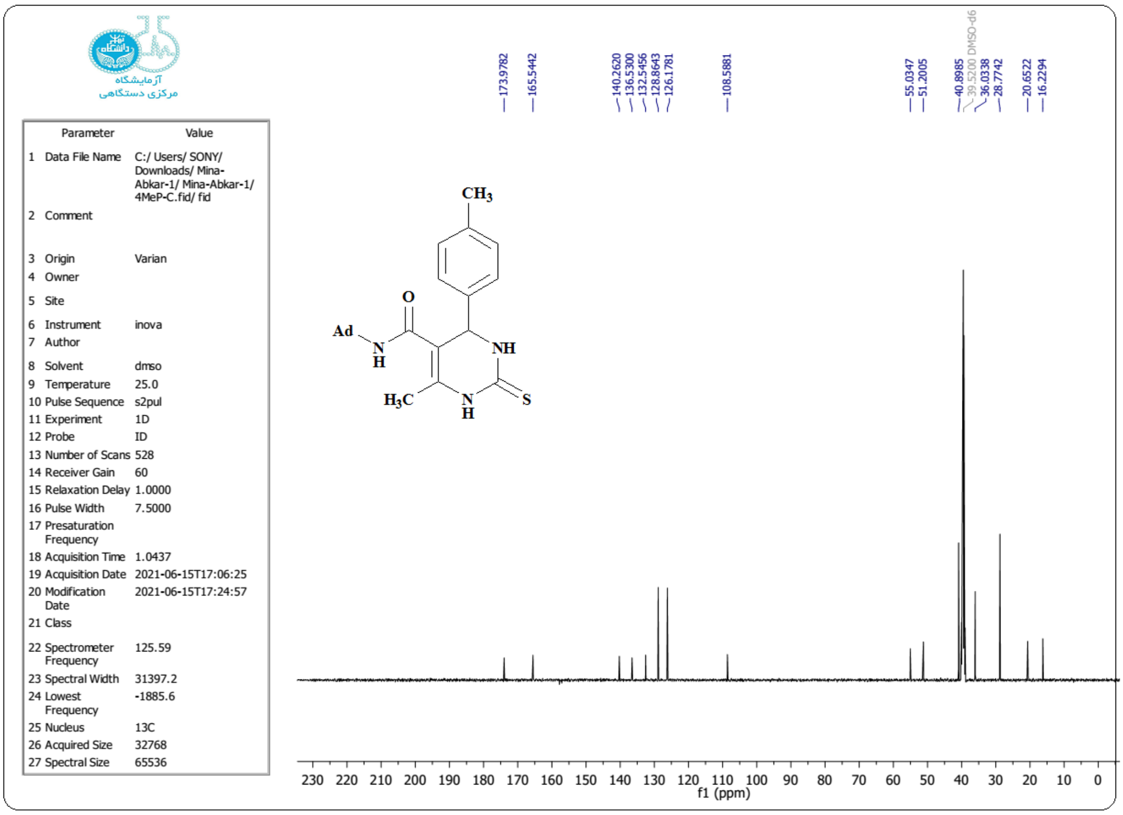

FTIR of 5-(1-adamantylcarbamoyl) −6-methyl-4-(3-methoxyphenyl) −3,4-dihydropyrimidin-2(1H)-thione [IIe]

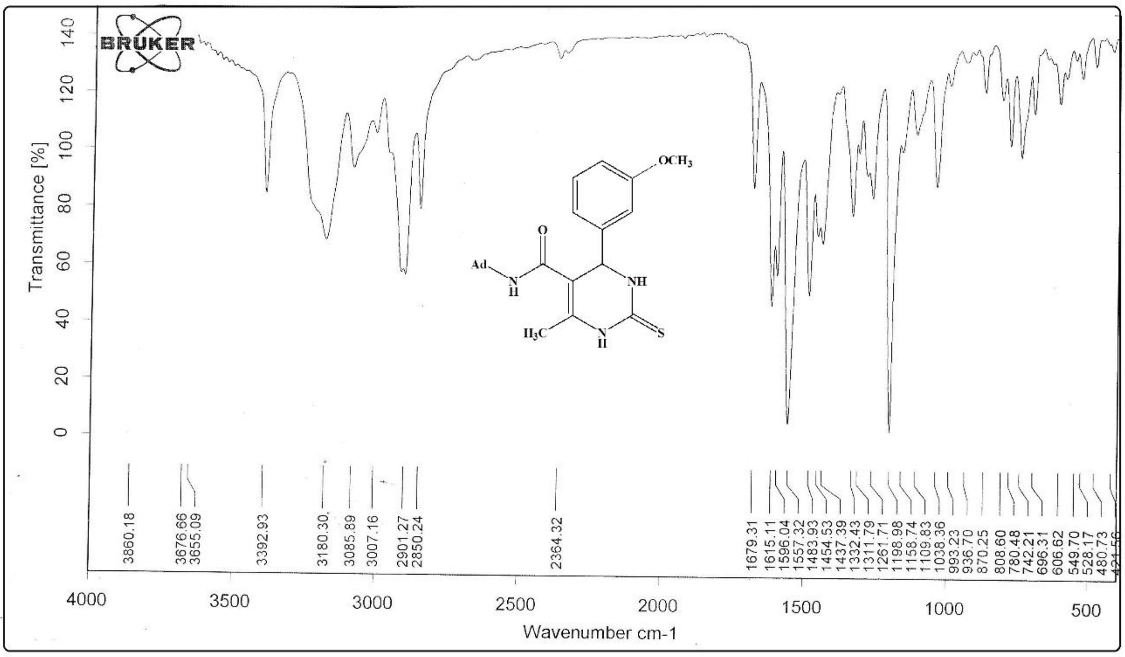

^{1}HNMR of 5-(1-adamantylcarbamoyl) −6-methyl-4-(3-methoxyphenyl) −3,4-dihydropyrimidin-2(1H)-thione [IIe]

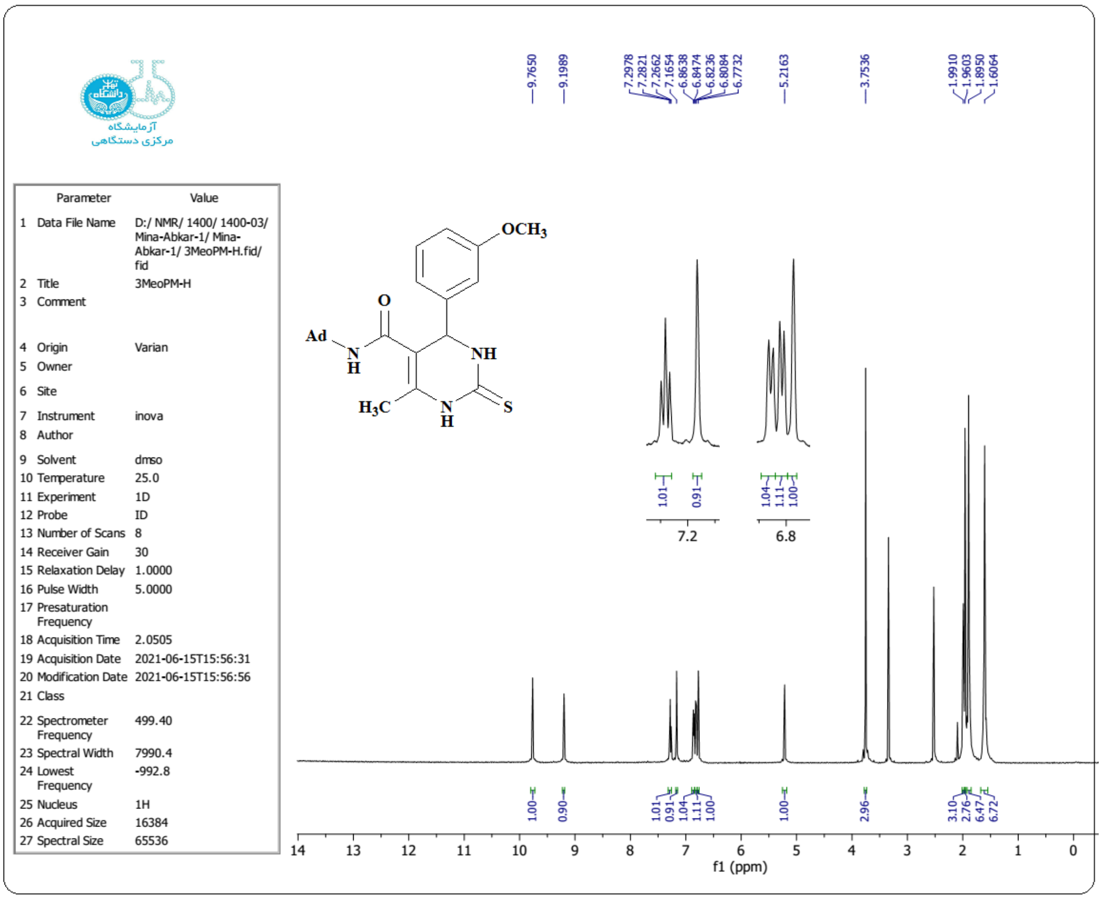

^{13}CNMR of 5-(1-adamantylcarbamoyl) −6-methyl-4-(3-methoxyphenyl) −3,4-dihydropyrimidin-2(1H)-thione [IIe]

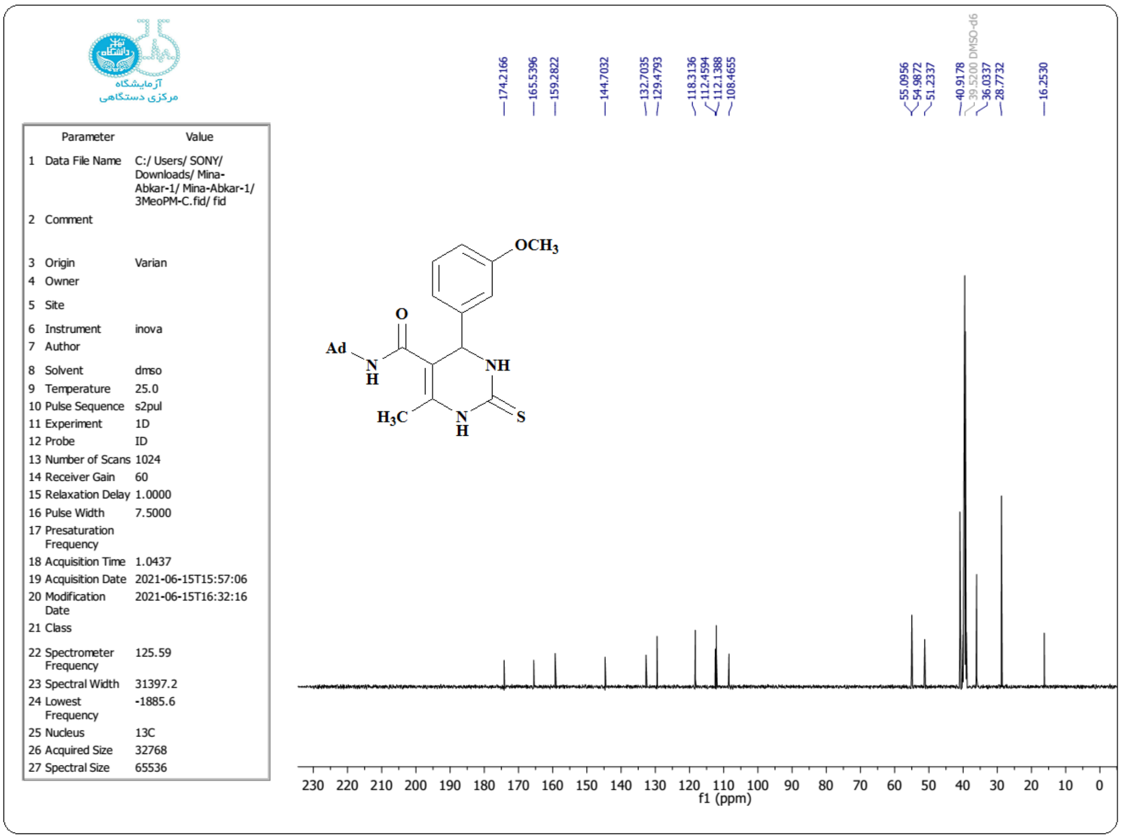

FTIR of 13-(1-adamantylcarbamoyl) −9-methyl-11-thioxo-8-oxa-10,12-diazatricyclo [7.3.1.0^{2,7}] trideca-2,4,6-triene [IIf]

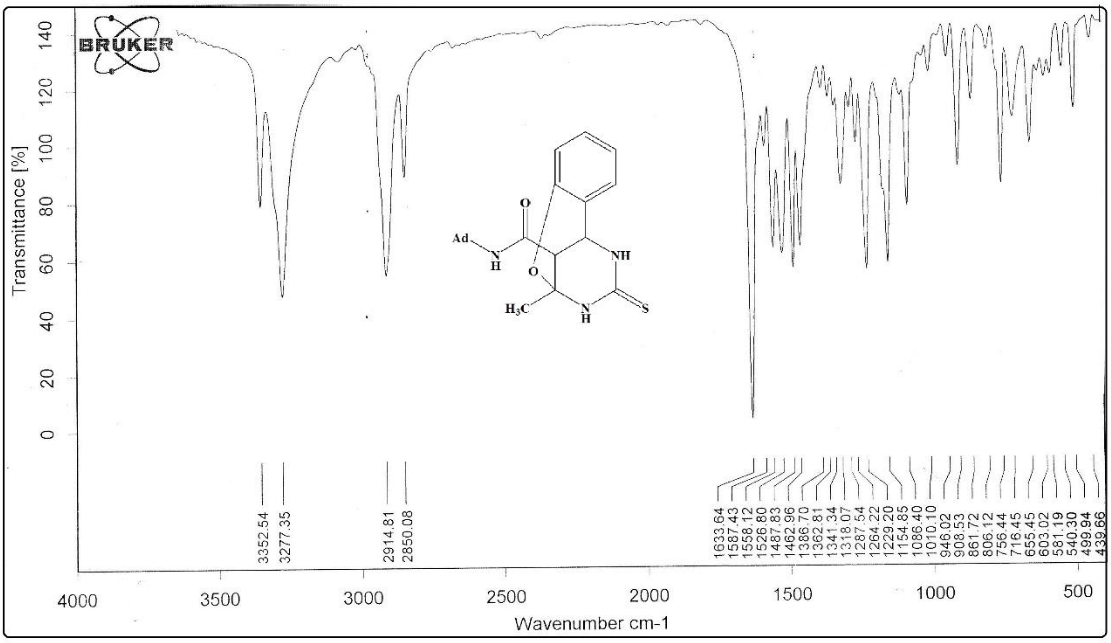

^{1}HNMR of 13-(1-adamantylcarbamoyl) −9-methyl-11-thioxo-8-oxa-10,12-diazatricyclo [7.3.1.0^{2,7}] trideca-2,4,6-triene [IIf]

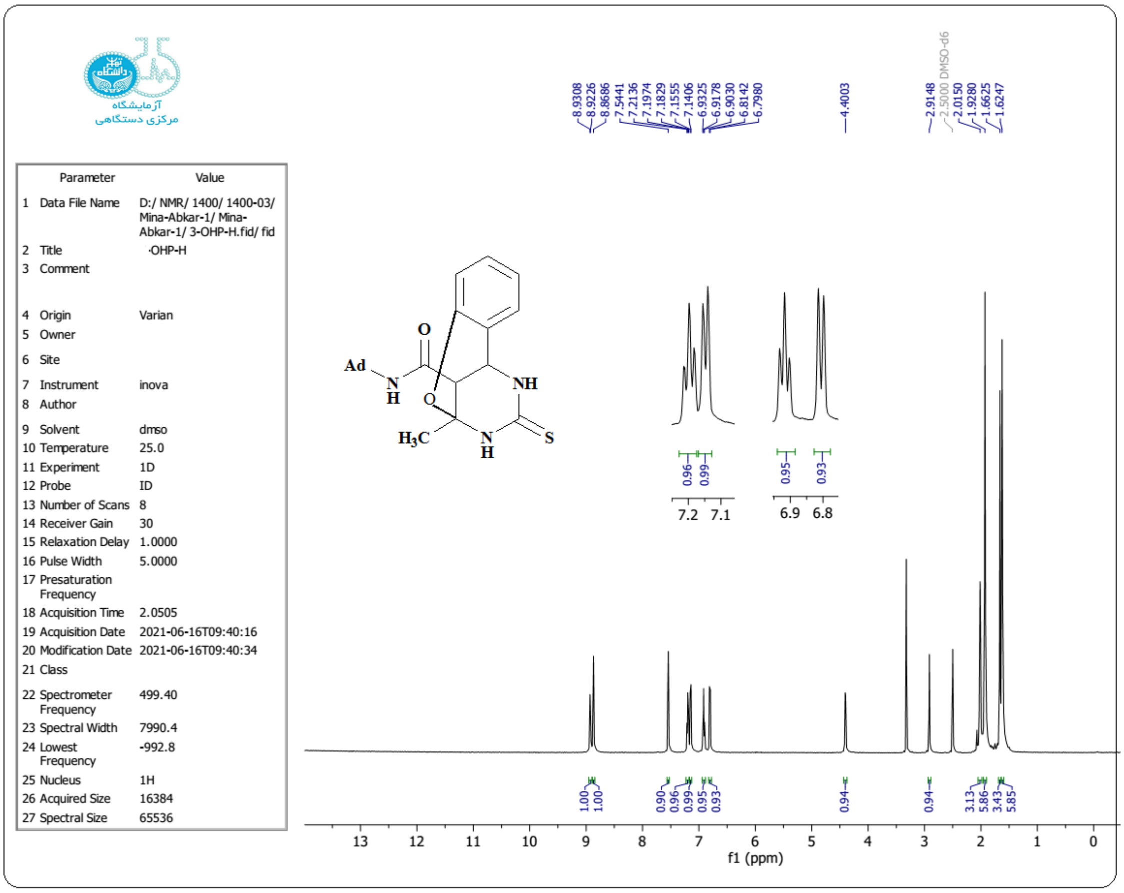

^{13}CNMR of 13-(1-adamantylcarbamoyl) −9-methyl-11-thioxo-8-oxa-10,12-diazatricyclo [7.3.1.0[2,7]] trideca-2,4,6-triene [IIf]

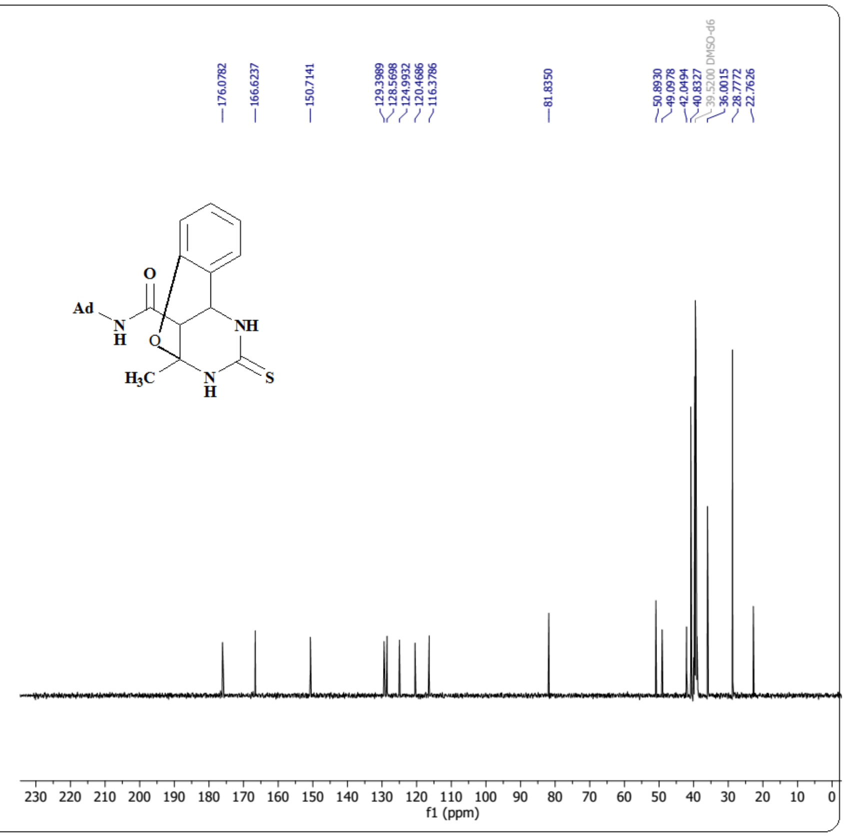

	Parameter	Value
1	Data File Name	D:/ NMR/ 1400/ 1400-03/ Mina-Abkar-1/ Mina-Abkar-1/ 3-OHP-C.fid/ fid
2	Title	-OHP-C
3	Comment	
4	Origin	Varian
5	Owner	
6	Site	
7	Instrument	inova
8	Author	
9	Solvent	dmso
10	Temperature	25.0
11	Experiment	1D
12	Probe	ID
13	Number of Scans	432
14	Receiver Gain	60
15	Relaxation Delay	1.0000
16	Pulse Width	7.5000
17	Presaturation Frequency	
18	Acquisition Time	1.0437
19	Acquisition Date	2021-06-16T09:40:39
20	Modification Date	2021-06-16T09:55:59
21	Class	
22	Spectrometer Frequency	125.59
23	Spectral Width	31397.2
24	Lowest Frequency	-1881.0
25	Nucleus	13C
26	Acquired Size	32768
27	Spectral Size	65536

FTIR of 5-(1-adamantylcarbamoyl)−6-methyl-4-(3-hydroxyphenyl)−3,4-dihydropyrimidin-2(1H)-thione [IIh]

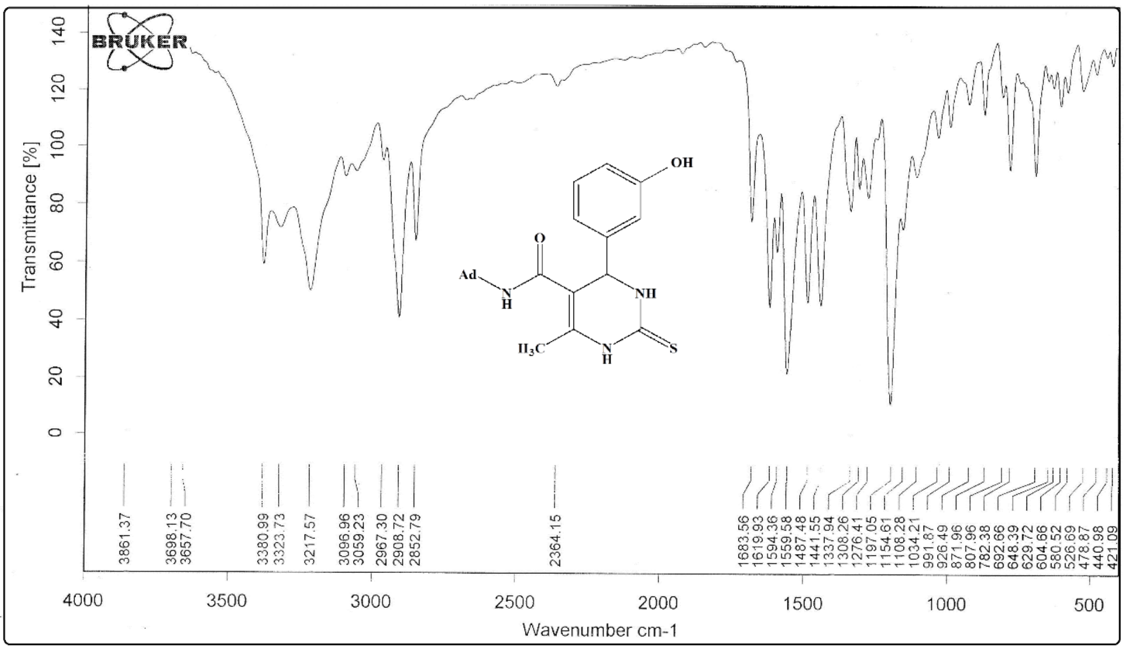

^{1}HNMR of 5-(1-adamantylcarbamoyl)−6-methyl-4-(3-hydroxyphenyl)−3,4-dihydropyrimidin-2(1H)-thione [IIh]

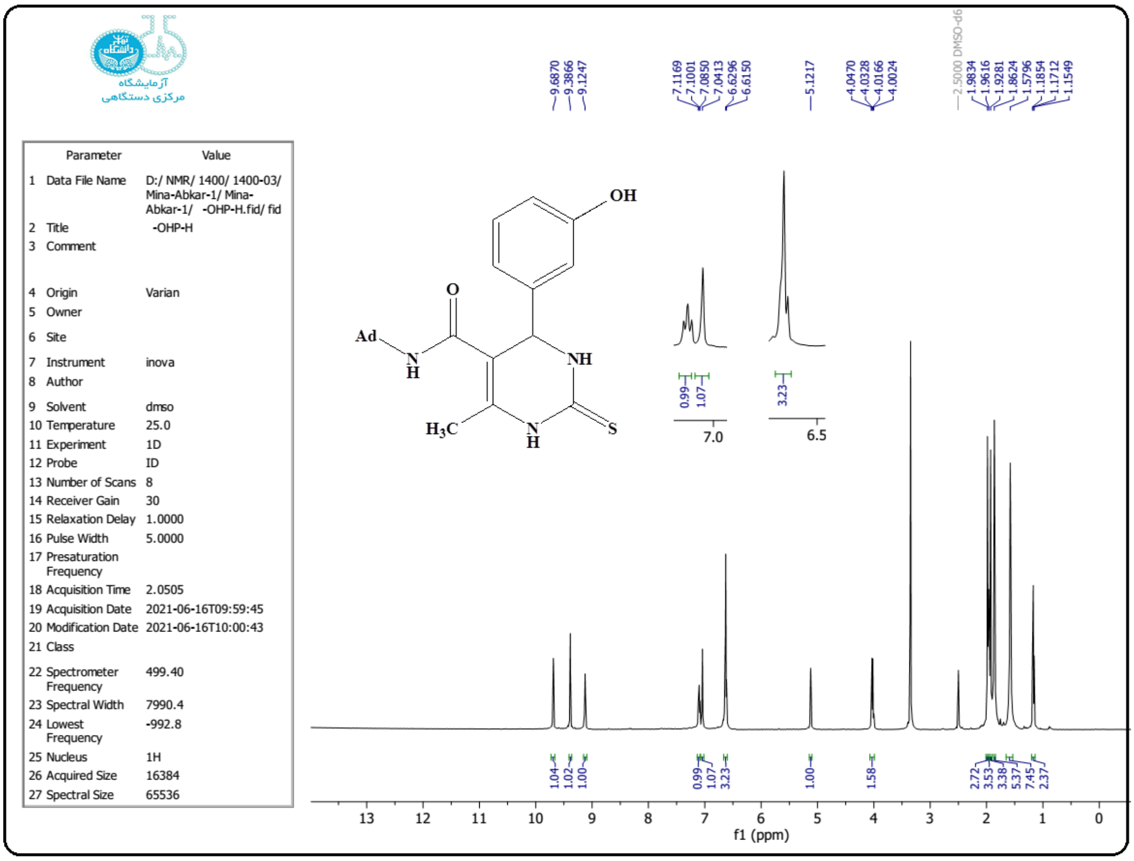

^{13}CNMR of 5-(1-adamantylcarbamoyl)−6-methyl-4-(3-hydroxyphenyl)−3,4-dihydropyrimidin-2(1H)-thione [IIh]

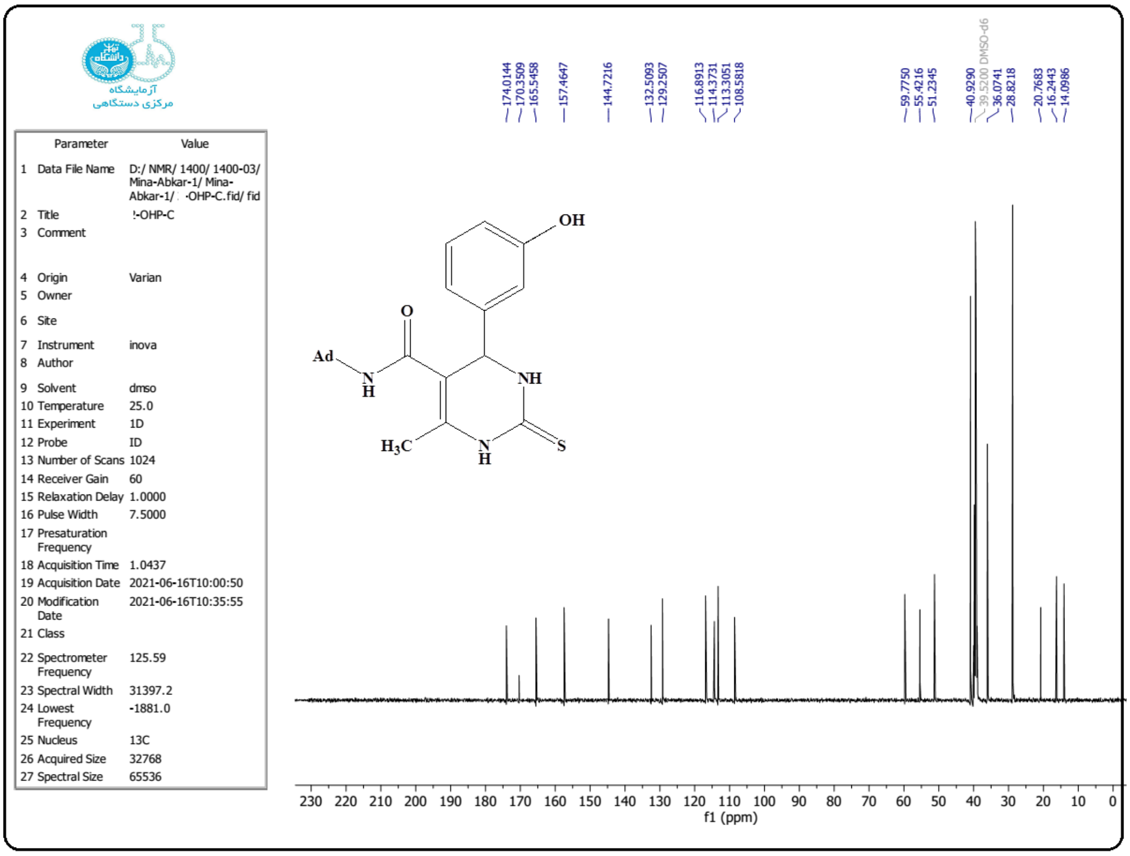

FTIR of 5-(1-adamantylcarbamoyl)−6-methyl-4-(3-nithrophenyl)−3,4-dihydropyrimidin-2(1H)-thione [IIi]

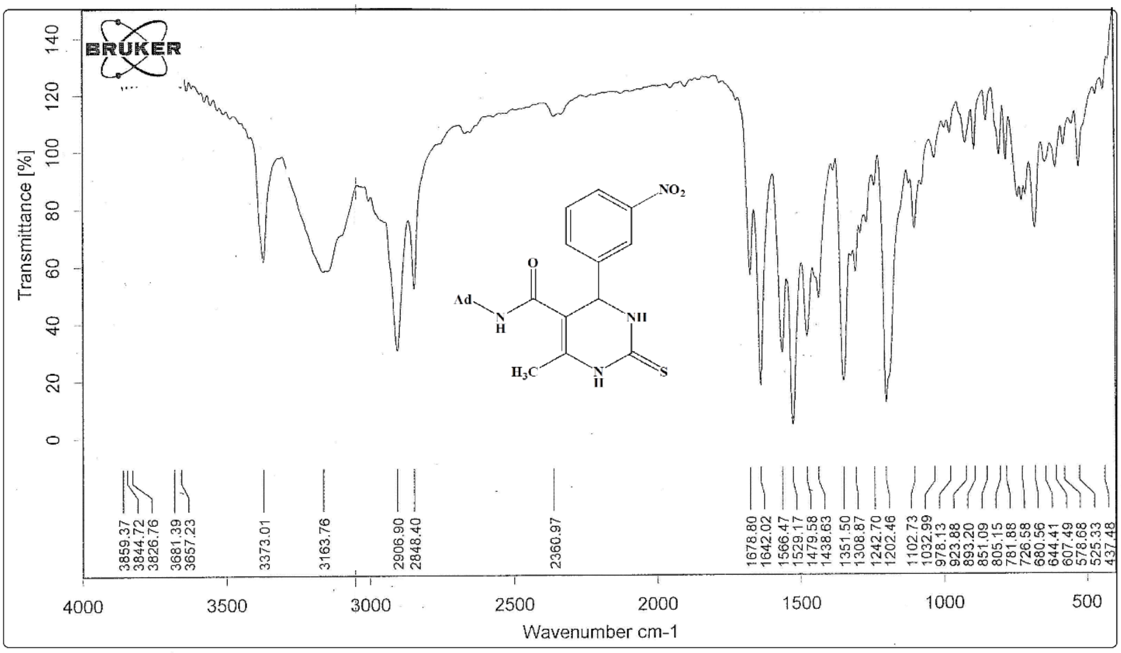

^{1}HNMR of 5-(1-adamantylcarbamoyl)−6-methyl-4-(3-nithrophenyl)−3,4-dihydropyrimidin-2(1H)-thione [IIi]

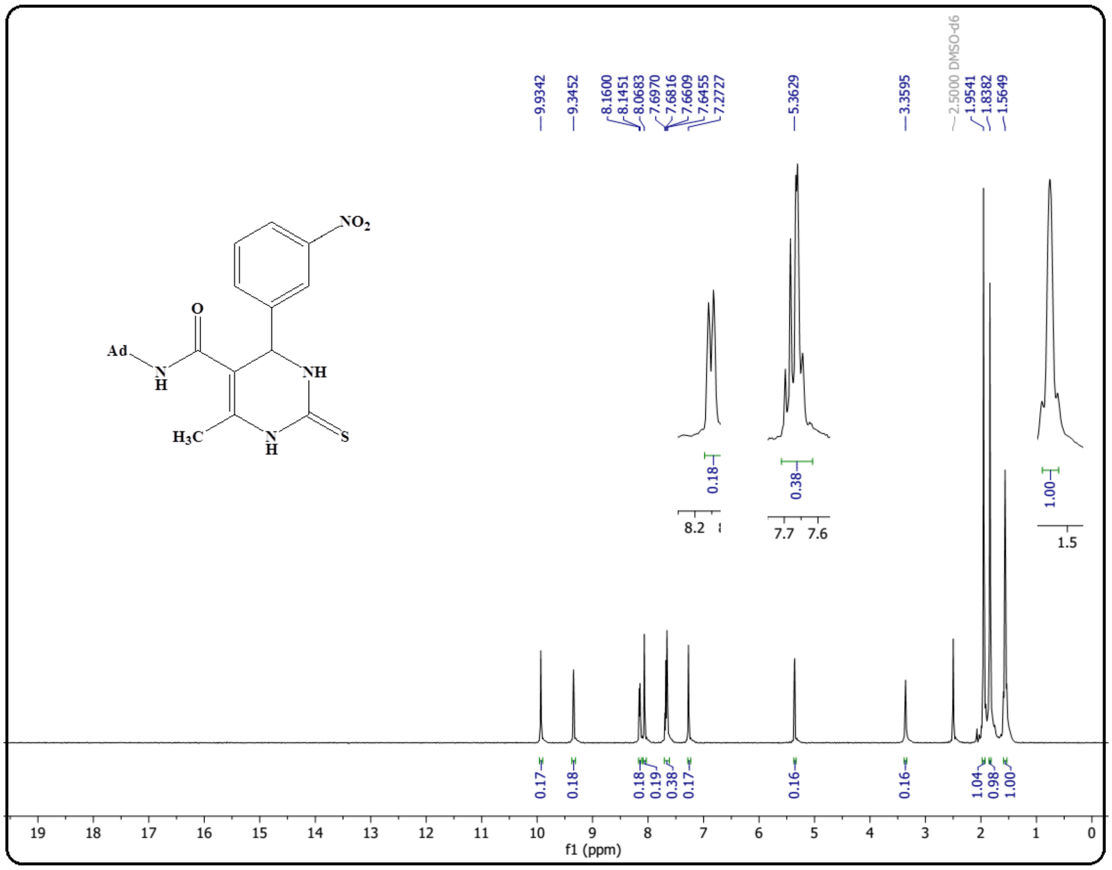

^{13}CNMR of 5-(1-adamantylcarbamoyl)−6-methyl-4-(3-nithrophenyl)−3,4-dihydropyrimidin-2(1H)-thione [IIi]

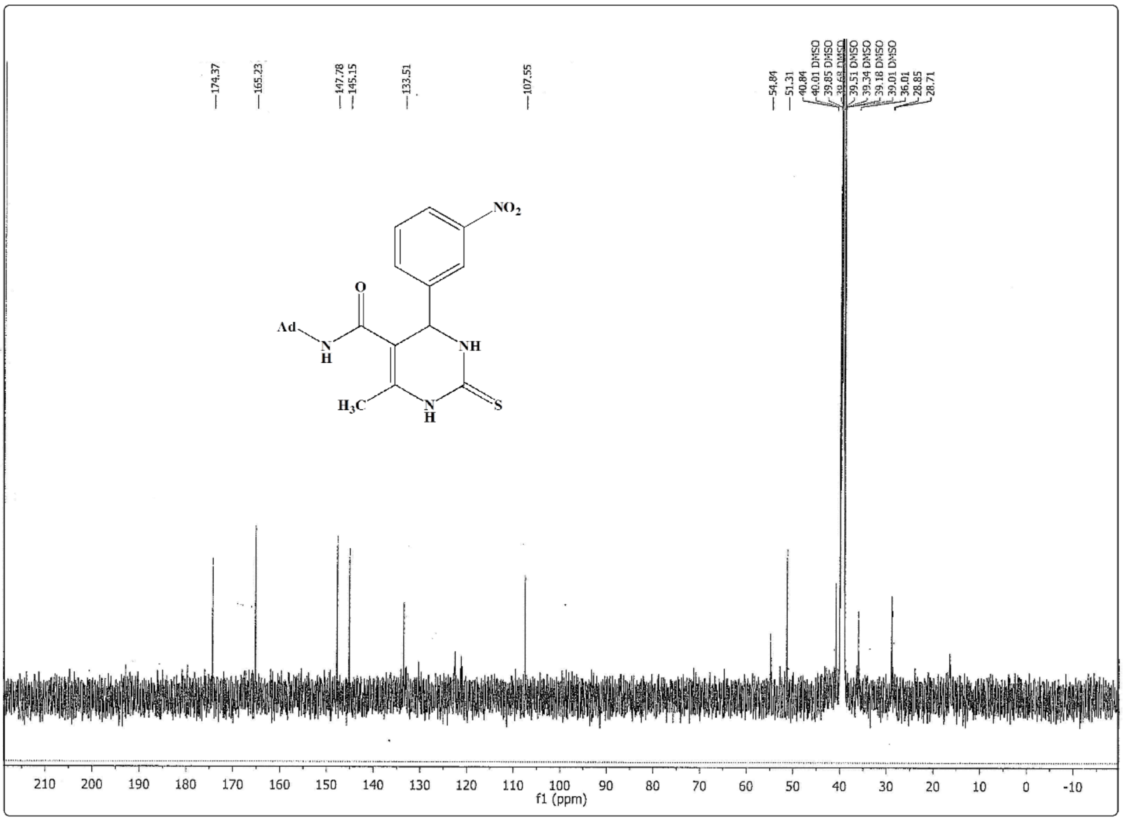

FTIR of 5-(1-adamantylcarbamoyl)−6-methyl-4-(4-nithrophenyl)−3,4-dihydropyrimidin-2(1H)-thione [IIg]

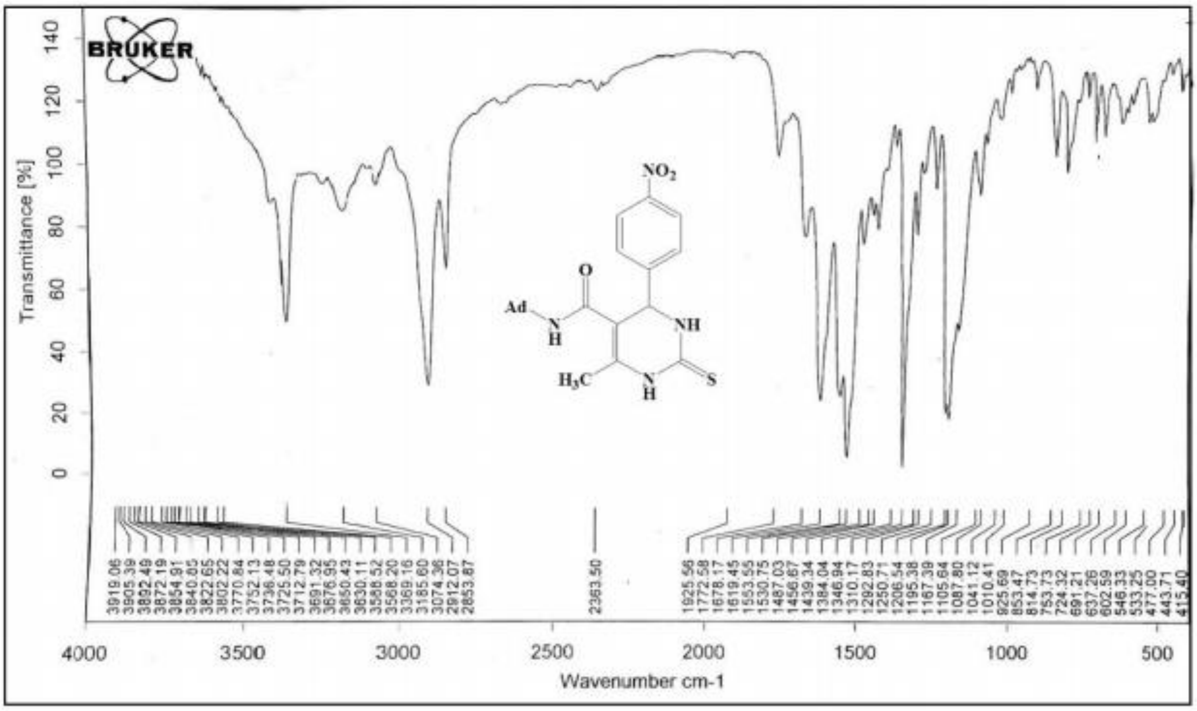

^{1}HNMR of 5-(1-adamantylcarbamoyl)−6-methyl-4-(4-nithrophenyl)−3,4-dihydropyrimidin-2(1H)-thione [IIg]

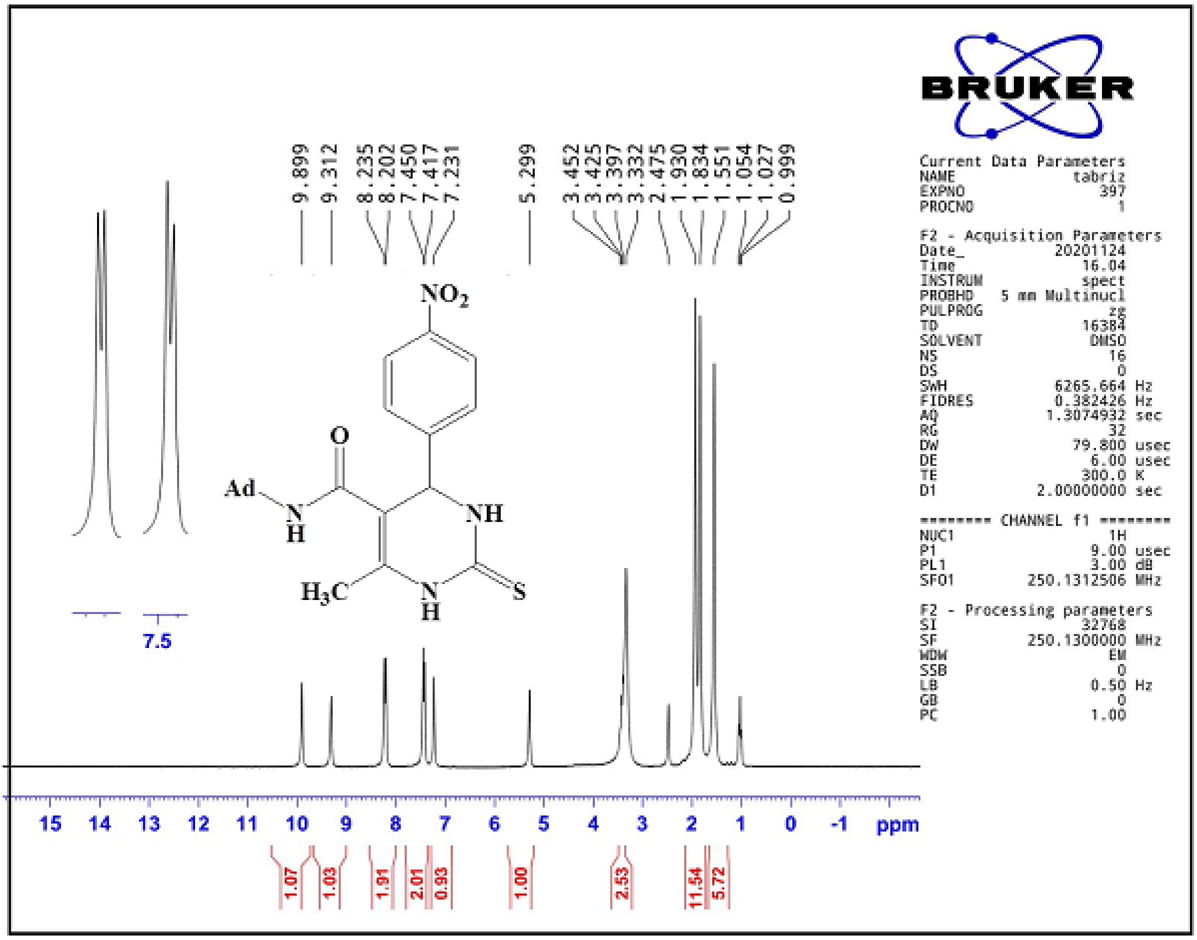

^{13}CNMR of 5-(1-adamantylcarbamoyl)−6-methyl-4-(4-nithrophenyl)−3,4-dihydropyrimidin-2(1H)-thione [IIg]

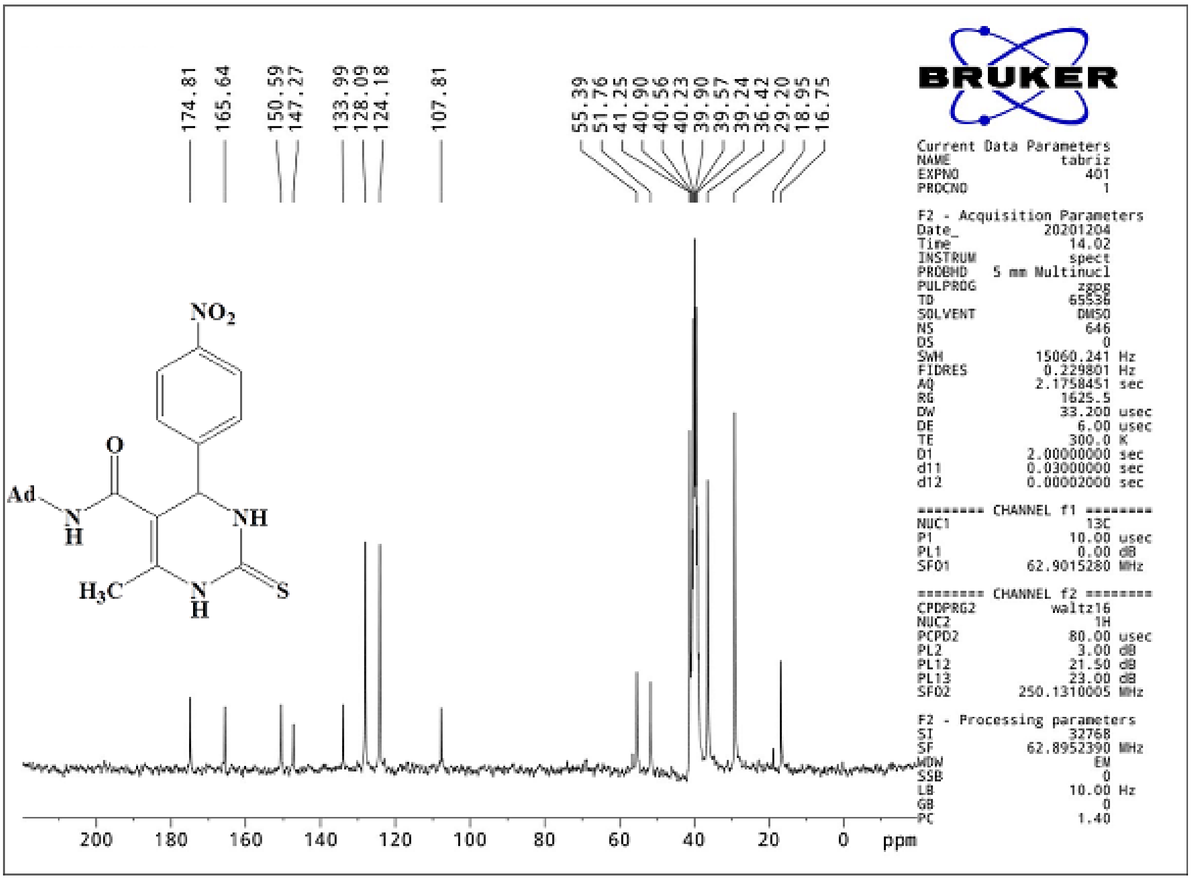

FTIR of 5-(1-adamantylcarbamoyl)−6-methyl-4-(2-chlorophenyl)−3,4-dihydropyrimidin-2(1H)-thione [IIj]

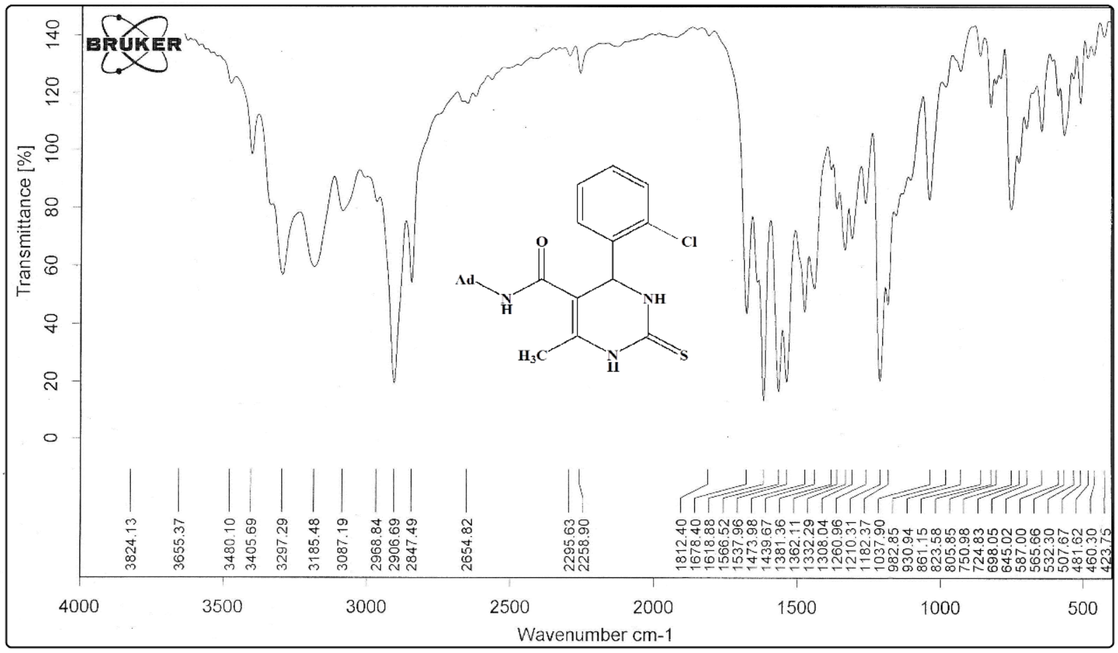

^{1}HNMR of 5-(1-adamantylcarbamoyl)−6-methyl-4-(2-chlorophenyl)−3,4-dihydropyrimidin-2(1H)-thione [IIj]

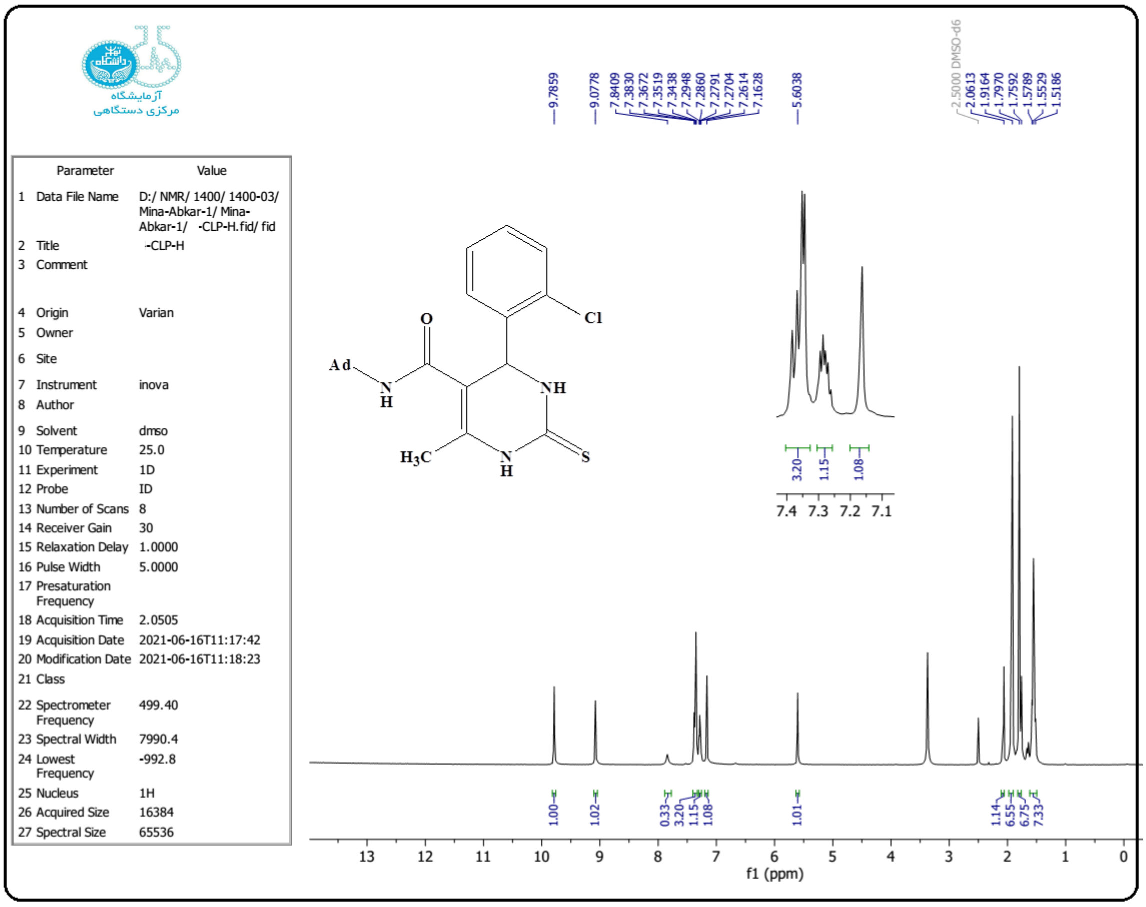

^{13}CNMR of 5-(1-adamantylcarbamoyl)‒6-methyl-4-(2-chlorophenyl)‒3,4-dihydropyrimidin-2(1H)-thione [IIj]

	Parameter	Value
1	Data File Name	C:/ Users/ SONY/ Downloads/ Mina-Abkar-1/ Mina-Abkar-1/ -CLP-C.fid/ fid
2	Comment	
3	Origin	Varian
4	Owner	
5	Site	
6	Instrument	inova
7	Author	
8	Solvent	dmso
9	Temperature	25.0
10	Pulse Sequence	s2pul
11	Experiment	1D
12	Probe	ID
13	Number of Scans	688
14	Receiver Gain	60
15	Relaxation Delay	1.0000
16	Pulse Width	7.5000
17	Presaturation Frequency	
18	Acquisition Time	1.0437
19	Acquisition Date	2021-06-16T11:18:29
20	Modification Date	2021-06-16T11:42:15
21	Class	
22	Spectrometer Frequency	125.59
23	Spectral Width	31397.2
24	Lowest Frequency	-1885.6
25	Nucleus	13C
26	Acquired Size	32768
27	Spectral Size	65536

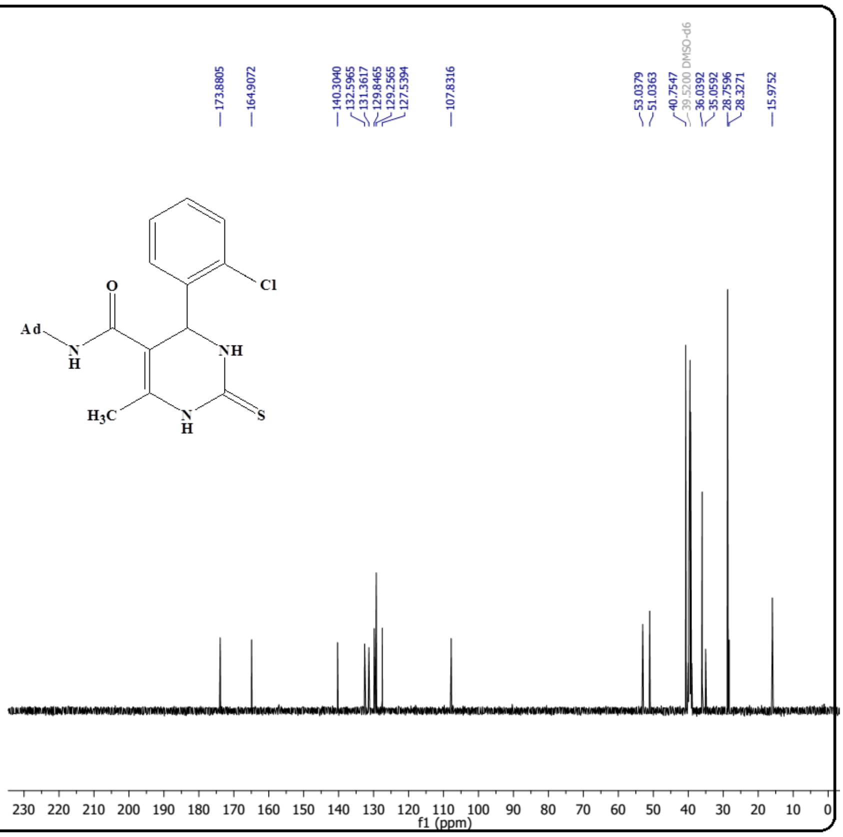

FTIR of 5-(1-adamantylcarbamoyl)−6-methyl-4-(3-chlorophenyl)−3,4-dihydropyrimidin-2(1H)-thione [IIk]

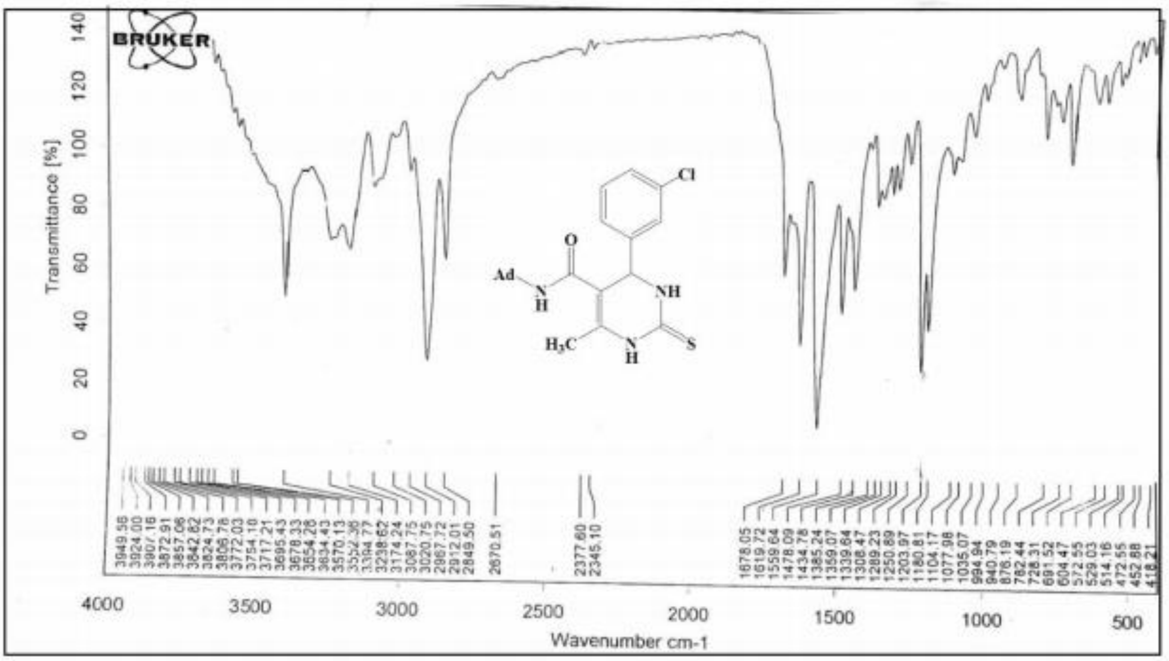

^{1}HNMR of 5-(1-adamantylcarbamoyl)−6-methyl-4-(3-chlorophenyl)−3,4-dihydropyrimidin-2(1H)-thione [IIk]

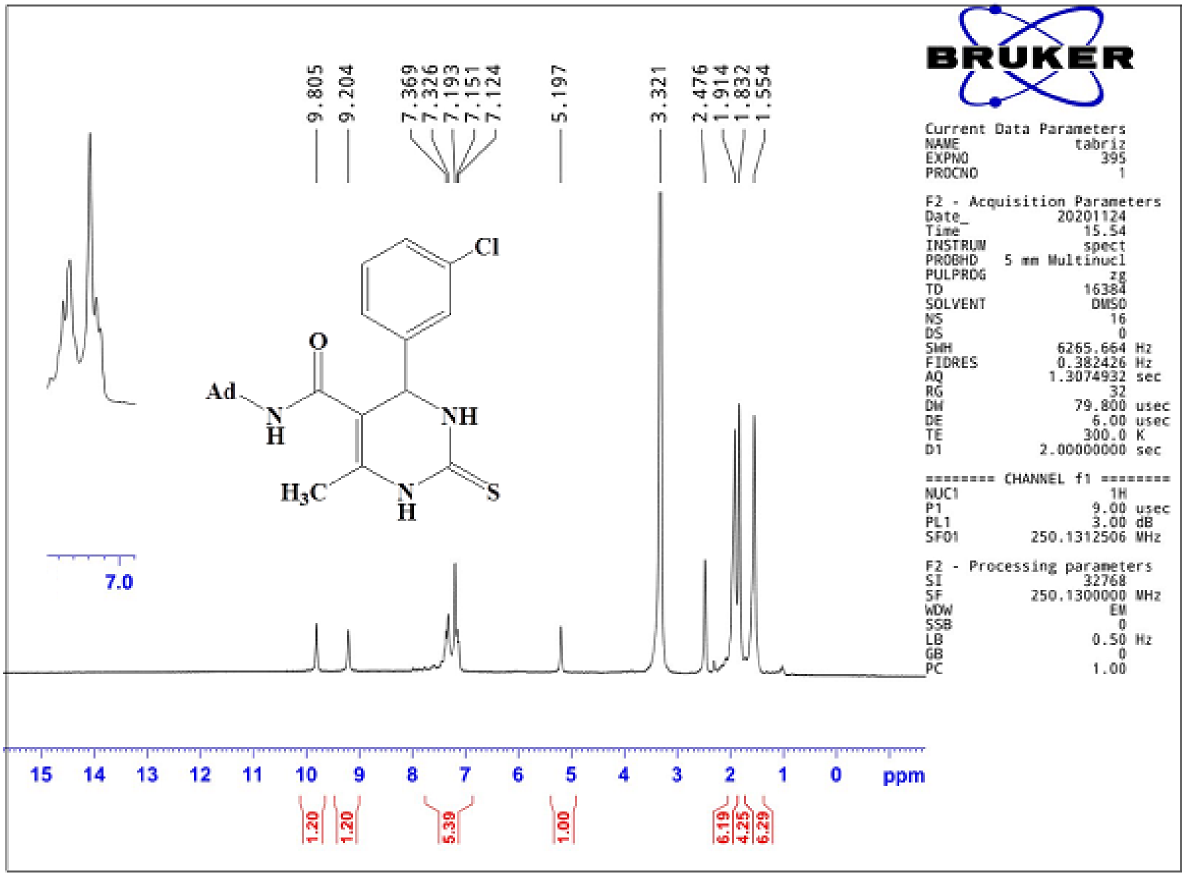

^{13}CNMR of 5-(1-adamantylcarbamoyl)−6-methyl-4-(3-chlorophenyl)−3,4-dihydropyrimidin-2(1H)-thione [IIk]

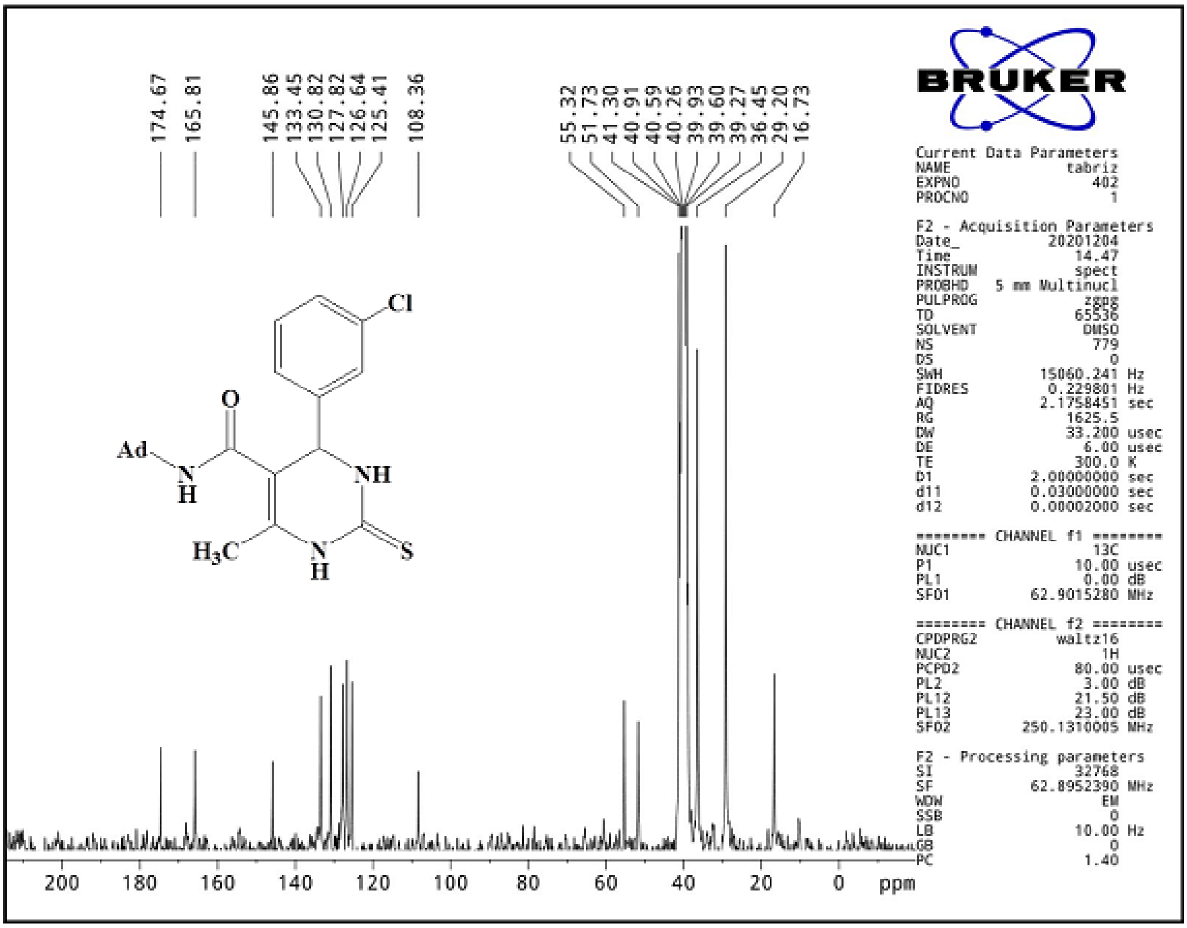

Supplement: RA-015-D5RA00284B-s001 [file RA-015-D5RA00284B-s001.pdf]
